# Supplementary material for: Machine Learning Prediction of Laccase‐Catalyzed Oxidation of Aromatic Compounds Using Curated Enzyme‐Specific Datasets
Source: J Comput Chem. 2026 Mar 6;47(7):e70344. doi: 10.1002/jcc.70344 (PMC12966443; doi:10.1002/jcc.70344)
Supplement: Supplementary file 1 — Data S1: Supporting Information. [file JCC-47-0-s001.pdf]

Machine learning prediction of laccase-catalyzed oxidation of aromatic compounds using curated enzyme-specific datasets

Yulia Kulagina[1,4*], Christian Goldhahn[2], Ramon Weishaupt[3], Mark Schubert[1]

[1]WoodTec, Swiss Federal Laboratories for Materials Science and Technology (Empa), Überlandstrasse 129, Dübendorf, CH-8600, Switzerland.
[2]Wood Materials Science, ETH Zürich, Stefano-Franscini-Platz 3, Zürich, CH-8093, Switzerland.
[3]Medical Department, A. Vogel AG, Grünaustrasse 4, Roggwil, CH-9325, Switzerland.
[4]Present address: Independent Researcher, Moscow, Russia. Author was affiliated with **1** during part of this work.

*Corresponding author(s). E-mail(s): jkulagina@protonmail.com;
Contributing authors: mark.schubert@empa.ch;

Abstract

Laccases are multi-copper oxidase enzymes that oxidize a wide range of aromatic and non-aromatic compounds using molecular oxygen, producing water as the sole byproduct and making them attractive biocatalysts for green chemistry. However, the ability of laccases to oxidize specific substrates depends on a complex interplay of molecular structure, enzyme properties, redox potential, and environmental context, making laccase–substrate compatibility hard to predict. We apply machine learning models to pre-screen laccase-substrate combinations, streamlining experimental workflows. We evaluate four classical classifiers and a transformer-based model (ChemBERTa) on three in-house curated datasets of aromatic substrates with oxidation profiles for distinct laccases. Overall, the tested models achieve comparable performance, with random forest (RFC) demonstrating more stability across different data splits and laccases. This assessment is complemented by RFC feature-importance and ChemBERTa attention analyses, which highlight molecular features associated with oxidation outcomes.

We also introduce a lightweight tool to visualize ChemBERTa predictions by mapping SMILES attributions onto molecular graphs. These findings provide a robust, interpretable framework for accelerating laccase–substrate discovery.

Keywords: Laccases, Biocatalysis, Machine learning, Chemical reactivity prediction, Molecular descriptors, SMILES, ChemBERTa

1 Introduction

1.1 Reaction mechanisms and applications of laccases

Laccases (EC 1.10.3.2) are multi-copper oxidase enzymes capable of oxidizing a wide range of aromatic and non-aromatic compounds, using molecular oxygen and producing only water as a byproduct. This environmentally friendly process makes laccases valuable as biocatalysts in industrial, environmental, and biotechnological applications, such as the synthesis of pharmaceuticals, textile bleaching, food preservation, biosensor development, etc. [1–7, 7–20].

Despite its potential, laccase-mediated catalysis remains underutilized due to the enzyme's complex substrate specificity, which depends on the interplay of molecular structure, enzyme properties, redox potential, and reaction conditions [13, 21, 22]. Consequently, identifying effective laccase-substrate pairs for specific applications remains a challenge, and typically relies on labor-intensive and costly experimental screening.

At the mechanistic level, laccase-catalyzed oxidation proceeds via radical-based one-electron transfer (SET). It involves electron abstraction by a copper center, generating phenoxy or aryl radical cations, which may subsequently undergo radical coupling ($C-C$ or $C-O$ bond formation), aromatic ring oxidation, or polymerization, while molecular oxygen is reduced to water through a four-electron transfer at the enzyme's copper cluster [22, 23].

1.2 ML methods in laccase-mediated oxidation prediction

Given the limitations of traditional experimental methods, machine learning (ML) provides a powerful alternative for predicting laccase-substrate compatibility. In this study, we explore five classification models – logistic regression with elastic net regularization (LogReg), support vector machines(SVC), random forests (RFC), gradient-boosted decision trees (GradBoostC), and a ChemBERTa transformer to predict the likelihood of aromatic substrates undergoing laccase-catalyzed oxidation. The models are trained and evaluated on three in-house curated datasets, comprising experimentally verified laccase-substrate oxidation profiles across distinct enzymes. These data represent a unique contribution to the field, enabling domain-specific model training and assessment not possible with generic chemical databases.

The rapid growth of computational power, accessible open-source chemical databases (e.g., ZINC, DrugBank, ChEMBL, PubChem) [24–27], and advances in ML algorithms have reshaped many fields, including molecular property prediction

(MPP), molecular design, chemical reaction prediction (CRP) and chemical engineering [28–33, 33–49]. While ML techniques are widely applied in these areas, their use in modeling enzymatic oxidation, particularly for laccases, is underexplored. This study aims to fill this gap, providing a foundational approach for more systematic and efficient enzyme-substrate matching.

1.3 Organization of the manuscript

The manuscript is organized as follows. Section 2 gives an overview of the data used in this work, including details of their curation. Section 3 describes the ML models and the rationale for their selection. Section 4 outlines the data-preprocessing steps and implementation workflows. Section 5 reports model results and interprets feature importance and model decisions. Section 6 discusses potential improvements and limitations of the employed techniques. The manuscript concludes with a summary of key findings and an outlook for future research.

2 Data

2.1 Data collection

Results on the oxidation of various aromatic substrate groups were obtained from experimental investigations conducted at the Swiss Federal Laboratories for Materials Science and Technology (Empa). The experiments followed the protocol described by Reiss et al. (2013) [21] and employed three laccase enzymes: Trametes versicolor (**f-tve**), Myceliophthora thermophila (**f-mth**), and Bacillus pumilus (**bpu-lac**).

The oxidation activity of the three laccases was systematically assessed using a panel of more than 250 structurally diverse small molecules. The experimental datasets contain 250, 190 and 172 records for the **f-tve**, **f-mth** and **bpu-lac** laccases, respectively. The fungal laccases (**f-tve** and **f-mth**) were purchased from Sigma-Aldrich and Novozymes, the bacterial enzyme (**bpu-lac**) was produced recombinantly in *Escherichia coli* transformed with a plasmid. Reactions were carried out in acidic buffer conditions (pH $= 6.0$) at $37°C$ with shaking at 100 rpm and monitored over 24 hours via UV-Vis spectroscopy ($230 - 700$ nm) to detect substrate oxidation through spectral changes. Each laccase-substrate pair was subsequently classified as follows:

(+) clear spectral change → substrate oxidized (Oxd $= 1$)
(−) no change → no oxidation (Oxd $= 0$)
(±) ambiguous/partial response.

These oxidation profiles provide a unique resource for modeling laccase-mediated oxidation, given their experimental origin and specificity to well-characterized enzymes – features often lacking in public databases. The oxidation profiles are derived from controlled, well-documented experimental assays, ensuring consistency across substrates and laccases. Each compound's reactivity was measured under standardized conditions, allowing for direct and reliable comparisons, which is an essential requirement for predictive modeling.

Furthermore, the data focus on three well-characterized laccases – **f-tve**, **f-mth**, and **bpu-lac**, with known structural and catalytic properties. This stands in contrast with many public datasets where oxidation annotations are often derived from heterogeneous sources, with little detail on enzyme identity, assay conditions, or substrate scope. The explicit enzyme-substrate mappings and quantitative oxidation outcomes enable more accurate structure–activity relationship modeling and ML applications, supporting better predictions of laccase reactivity and more rational enzyme engineering.

2.2 Datasets overview

Despite the narrow scaffold space, the datasets show high substituent-level and physicochemical diversity, which is captured in the ECFP4[1] similarity metrics [50]: mean Tanimoto similarity [51] is low across all datasets ($0.167 - 0.186$), with high internal diversity values ($0.814 - 0.833$). The nearest-neighbor similarity distributions consistently peak around 0.55–0.65, indicating the presence of local analog series but also a substantial number of structurally distinct substituent patterns. UMAP (Uniform Manifold Approximation and Projection) projections [52] reinforce this: the chemical space forms a single, dense region corresponding to substituted benzene derivatives, but is horizontally broadened by varied substituent types and patterns (see Figure S1).

The top 3 scaffolds cover 80–87% of each dataset, indicating that while various functional classes (alcohols, ketones, aldehydes, amines) are present, they tend to rely on the same aromatic core [53]. Across all three datasets, benzene (*c*1*ccccc*1) dominates the scaffold distribution. This is expected for laccase-relevant chemistry, but it materially constrains out-of-distribution generalization: the models are primarily trained to discriminate substrates within a narrow benzenoid scaffold family, and predictions for heteroaromatic, fused-ring, or otherwise non-benzenoid scaffolds are therefore not well supported by the present data.

The Jaccard analysis also shows high scaffold overlap ($J = 1.0$) among most functional groups. The high scaffold concentration and near-complete scaffold overlap across functional groups imply that much of the apparent functional diversity occurs as variations of substituents on the same aromatic core. Consequently, the models are expected to generalize more reliably within benzenoid analog series (i.e., to unseen substituent patterns on the benzene scaffold) than across scaffold classes. In practice, this means that good performance on random splits may partly reflect interpolation among close analogs, whereas true extrapolation to underrepresented scaffold types (heteroaromatics, polycyclic systems, non-aromatics) remains uncertain.

Overall, the datasets offer good functional and electronic diversity but limited structural breadth. This pattern is typical for real-world laccase substrate collections, where benzenoid aromatics dominate experimental use. ML models trained on these data can still learn generalizable mechanistic features such as substituent electronics, steric effects, and radical stability, but their ability to extrapolate to non-benzenoid or underrepresented aromatic scaffolds remains constrained. Expanding the datasets with heteroaromatic and fused-ring systems would enhance scaffold diversity, improve feature robustness, and strengthen predictive power across a broader chemical space.

[1] ECFP4 = Extended-Connectivity Fingerprints with a diameter of 4 bonds.

2.3 Chemical structure representations

Concise, unambiguous molecular representations, understandable for both humans and machines, remain a central challenge in chemoinformatics. Molecules with multiple functional groups, diverse ring systems, nonstandard valencies, inorganic components, branching, chirality, or symmetry, often lead to non-unique encodings or collisions [54].

Numerous methods of molecular featurization have been suggested over the past years [30, 31, 54–56]: ECFPs [50], connection tables [57, 58], feature-based representations, 2D/3D molecular graphs [58–60], string representations, such as SMILES and SMARTS [61, 62], computer-learned representations, such as SELFIES[2] [63], etc. Each approach reflects the trade-offs between expressiveness, uniqueness, and robustness [54].

We characterized substrate structure and physicochemical properties using a broad set of molecular descriptors computed with the DRAGON software suite [64], a widely adopted tool for quantifying features relevant to reactivity prediction. The resulting descriptors span constitutional metrics (e.g., rotatable bonds, molecular weight, heteroatom count), topological indices (electrotopological state, pseudoconnectivity), ring parameters (detour ring indices), functional group counts, and P-VSA–type descriptors integrating van der Waals surface areas with atomic properties. This diverse feature set enables statistical models to capture subtle patterns influencing oxidation outcomes, even under high structural variability.

Each substrate was also encoded as a SMILES string, a human-readable linear notation that preserves connectivity, aromaticity, chirality, ring closures, and charge states. Canonical SMILES were generated by submitting IUPAC names to the Wolfram—Alpha REST API, ensuring standardized representations suitable for machine-learning models, including string-based architectures such as ChemBERTa.

2.4 Representation strategy used in the paper

To balance human interpretability, machine compatibility, and chemical expressiveness, we selected two complementary representations: molecular descriptors for classical ML and SMILES strings for sequence-based deep learning. This strategy enables comparison of feature-engineering with representations learning, while ensuring comprehensive coverage of chemical information relevant to oxidation prediction.

3 Methods

We investigate two model families for predicting laccase-mediated oxidation (see Figure 1): descriptor-based models using classical classifiers and a fine-tuned SMILES-based Transformer model.

Our goal is to develop a simple, flexible ML framework for robust classification of substrate oxidation in the presence of a given laccase. Formally, we aim to learn a

[2]SMILES = Simplified Molecular Input Line Entry System, SMARTS = SMiles ARbitrary Target Specification, SELFIES = SELF-referencing Embedded String.

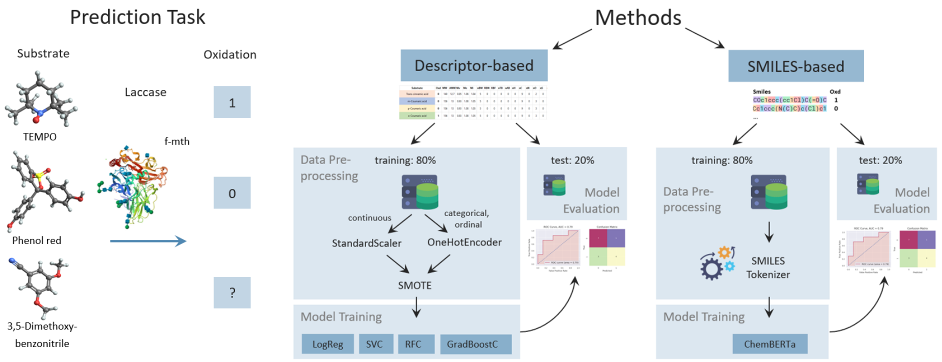

Figure 1 Prediction task and methods overview

function

$$f : \mathcal{X} \to \mathcal{Y}$$
$$\mathbf{x} \mapsto y,$$

where $\mathbf{x}$ represents either chemical descriptors $\mathbf{x} \in \mathcal{X} \subseteq \mathrm{R}^p$, with $p \in \mathbf{N}_+$ denoting the number of descriptors or SMILES-encoded features, and $y \in \mathcal{Y} = \{0, 1\}$ indicates whether the substrate undergoes oxidation.

Given a training dataset with n examples, $\mathcal{D} = \{(\mathbf{x}_i, y_i)\}_{i=1}^n$, where $\mathbf{x}_i \in \mathbb{R}^p$ represents the feature vector for the i-th example and $y_i \in \mathcal{Y}$ corresponding to the label, we would like to find parameter vector $\theta^\star$ s.t. f_θ minimizes the empirical risk [65] over the class of functions $\mathcal{F}$:

$$\min_{f \in \mathcal{F}} \frac{1}{n} \sum_{i=1}^{n} \mathcal{L}\big(y_i, f_\theta(\mathbf{x}_i)\big), \tag{1}$$

where the loss function $\mathcal{L}$ measures the discrepancy between the predicted and true labels for each data point.

We include four complementary classifiers to trade off bias, variance, and interpretability. Elastic-net logistic regression (LogReg) minimizes penalized log-loss with a convex combination of $L1$ and $L2$ penalties, where the $L1$ term encourages sparsity and variable selection under collinearity, while $L2$ stabilizes coefficients. Support vector classification (SVC) maximizes the margin using the hinge loss. We employ linear and RBF kernels to capture nonlinear decision boundaries, with the C parameter controlling the fit–margin trade-off. Random forests (RFC) aggregate multiple bootstrapped, decorrelated trees split by Gini impurity criterion, reducing variance via bagging and offering robust performance. Gradient-boosted trees (GradBoostC) add shallow trees stage-wise to correct residuals, controlling complexity through learning rate, depth, and subsampling. For all classical models, class imbalance is handled via oversampling of the minority class. Model-specific objectives for solving (1) (log-loss, hinge, impurity, boosting losses) follow standard formulations and are omitted for brevity. Readers interested in full objective definitions are invited to consult the Appendix.

ChemBERTa [66, 67] is a transformer specifically adapted for chemistry and leverages self-supervised learning to extract meaningful structural and semantic features from SMILES, and is built on the RoBERTa architecture [68]. For binary classification, we use the weighted binary cross-entropy (BCE) loss [69] to correct for class imbalance:

$$\mathcal{L}\big(y_i, f_\theta(\mathbf{x}_i)\big) = -\frac{1}{n}\sum_{i=1}^{n}\Big[w_1 y_i \log f_\theta(\mathbf{x}_i) + w_0(1-y_i)\log(1-f_\theta(\mathbf{x}_i))\Big], \tag{2}$$

with class weights $w_1 = \frac{n}{2n_1}$ and $w_0 = \frac{n}{2n_0}$, and n_1 and n_0 are positive and negative sample counts. The model output $f_\theta(\mathbf{x}_i)$ is the sigmoid-activated predicted probability.

ChemBERTa employs a byte-pair encoding (BPE) tokenizer, trained on chemical corpora, to convert raw SMILES into tokens – shorter, information-rich units that often correspond to common chemical substructures or motifs. These tokens are embedded, yielding an input matrix $\mathbf{E} \in \mathbb{R}^{l\times d}$which is linearly projected into query ($\mathbf{Q}$), key ($\mathbf{K}$), and value ($\mathbf{V}$) matrices using the learned weight matrices:

$$\mathbf{Q} = \mathbf{E}\mathbf{W}_Q, \mathbf{K} = \mathbf{E}\mathbf{W}_K, \mathbf{V} = \mathbf{E}\mathbf{W}_V,$$

where $\mathbf{W}_Q, \mathbf{W}_Q, \mathbf{W}_V \in \mathbb{R}^{d\times d_k}$, and $d_k = \frac{d}{h}$ denotes the dimensionality of each attention head for h attention heads[3].

A 6-layer, 12-head transformer encoder then applies scaled dot-product self-attention mechanism, allowing the model to dynamically focus on the most relevant parts of the input sequence by weighting token interconnections according to their contextual importance:

$$\text{Attention}(\mathbf{Q}, \mathbf{K}, \mathbf{V}) = \text{softmax}\left(\frac{\mathbf{Q}\mathbf{K}^T}{\sqrt{d_k}}\right)\mathbf{V}. \tag{3}$$

The training involves two stages. Pre-training uses masked language modeling (MLM), where a fraction of tokens is masked and predicted from context by minimizing the negative cross-entropy:

$$\mathcal{L}_{\text{MLM}} = -\frac{1}{|\mathcal{M}|}\sum_{i\in\mathcal{M}} \ln P(\text{token}_i|\text{context}), \tag{4}$$

where $\mathcal{M}$ denotes the set of masked token positions. This encourages the model to learn general-purpose representations of molecular structures. Fine-tuning uses multitask regression (MTR) to predict multiple molecular properties simultaneously:

$$\mathcal{L}_{\text{MTR}} = \frac{1}{N}\sum_{i=1}^{n}\sum_{j=1}^{g}\big(f_\theta(\text{SMILES}_i) - y_{ij}\big)^2, \tag{5}$$

[3]The notation assumes scaled dot-product attention and that attention heads are concatenated before being passed to downstream layers.

where n is the number of molecules in the training dataset, g is the number of target properties (tasks), $f_\theta(\text{SMILES})_i$ is the predicted value of property j for example i, y_{ij} is the corresponding ground truth.

Through this pipeline, ChemBERTa acquires chemically meaningful representations stored in its model weights, enabling accurate and data-efficient molecular property prediction.

4 Implementation details

4.1 Software details

Classical models were implemented using sklearn [70] via the `LogisticRegression()`, `SVC()`, `RandomForestClassifier()`, and `GradientBoostingClassifier()` classes, with hyperparameters optimized using cross-validation (CV) via `GridSearchCV()`. For ChemBERTa, we used the Transformers library [71] to access model configurations, tokenizers, and pretrained weights, and PyTorch [72] to optimize the loss defined in (2). Hyperparameter tuning was performed with Optuna [73]. Parameter grids and code are available in the project repository: https://github.com/empa-woodtec/laccase-oxd.

4.2 Data preprocessing

From an initial pool of over 3000 descriptors, we selected 70–90 per dataset (depending on the dataset). Features dominated by a single value (relative frequency > 0.95) were removed to eliminate redundancy and reduce noise.

The **f-tve** dataset shows significant class imbalance, with 74% positive samples. In contrast, the **f-mth** and **bpu-lac** datasets are nearly balanced, containing 54% and 53% positives, respectively. Since imbalance can degrade model robustness and generalization, we address it using method-specific strategies. For the classical ML models trained on descriptors, we apply the borderline synthetic minority over-sampling technique (Borderline-SMOTE) [74, 75] to oversample the minority class in the training subset only (via `BorderlineSMOTE()` in imbalanced-learn [76]). For ChemBERTa, we do not oversample, instead, we account for class imbalance by using a class-weighted binary BCE loss (2) during training.

4.3 Implementation of descriptor-based models

With the descriptor-based representation we evaluate four classical ML classifiers: LogReg, SVC, RFC, GradBoostC. The selection of these models is motivated by several practical considerations: limited training data, high-dimensional feature space, mixed nature of the descriptors (continuous, ordinal, and categorical), and the need for computational efficiency and interpretability. Under such conditions, classical ML methods often outperform more complex deep architectures [77–80].

Our descriptors include heterogeneous feature types. Tree-based models (RFC, GradBoostC) handle this naturally, whereas LogReg and SVC require explicit preprocessing for stable convergence. To ensure robustness and assess preprocessing sensitivity, we test two strategies: (a) standard scaling for continuous descriptors + one-hot encoding (OHE) for ordinal and discrete features, versus (b) min-max scaling

for continuous features + coarse binning with subsequent label-encoding for high-cardinality discrete variables. Both strategies produce comparable performance (Table ST1). For the remainder of this work, we adopt strategy (a).

Hyperparameters for all four classifiers are tuned using 5-fold CV. Final binary predictions are obtained using a 0.5 probability threshold. In practice this value may be adjusted based on application-specific priorities (to minimize false positives (FP) or false negatives (FN), depending on which type of error is more critical) in the downstream application.

4.4 Fine-tuning of the ChemBERTa model

We fine-tune ChemBERTa on the laccase data by retaining the pretrained transformer attention layers (backbone) and replacing the original regression head with a dropout layer to mitigate overfitting [81], followed by a dense classification layer. During fine-tuning, the MTR objective (5) is replaced by (2).

We explore two fine-tuning strategies: backbone freezing, where only the dropout and classification head are updated, while the backbone remains frozen and full fine-tuning, where all model parameters, including the attention backbone, are updated via backpropagation. Since the former strategy yields substantially lower performance, all reported results use full fine-tuning. Intermediate strategies (e.g., partial layer freezing) were not explored but may be promising for future work.

To ensure comparability with descriptor-based models, we use the same train–test split sample indices. We evaluated two Hugging Face ChemBERTa variants: ChemBERTa-zinc-base-v1[4] (pretrained on ∼250K ZINC15 molecules) and PubChem10M_SMILES_BPE_450k[5] (pretrained on ∼10M PubChem SMILES). We report results with PubChem10M_SMILES_BPE_450k, selected for its broader chemical coverage beyond drug-like space.

Fine-tuning uses 5-fold CV for hyperparameter optimization. The tuned parameters include: `batch_size`, `learning_rate`, `dropout_rate`, and `patience` (for early stopping), with the AdamW optimizer used for training. The `patience` parameter controls early stopping based on both validation loss and validation F1 score, helping prevent overfitting while improving discrimination between oxidized and non-oxidized substrates.

4.5 Evaluation protocol

We performed a nested CV procedure to ensure robust model evaluation. The process can be described as follows:

1. Train/test split: for each dataset, we used an 80% training and 20% test splits.
2. Inner cross-validation for hyperparameter tuning: within the 80% training set, we performed 5-fold CV to optimize the hyperparameters of each model. The training data were further divided into 5 folds: one fold was used as a validation set to tune the model, while the remaining 4 folds were used to train the model.

[4] https://huggingface.co/seyonec/ChemBERTa-zinc-base-v1
[5] https://huggingface.co/seyonec/PubChem10M_SMILES_BPE_450k

3. Outer evaluation: the final model, trained on the entire 80% training set (after hyperparameter optimization), was evaluated on the held-out 20% test set.
4. Repetition for stability: to ensure the results were not overly sensitive to a particular data split, we repeated the entire process 10 times with different random seeds. Each repetition used a new random 80/20 split to further improve the reliability and generalization of the model performance.
5. Performance metrics: the final results reported were obtained by averaging the performance metrics (accuracy, precision, recall, F1 score, AUROC) across these 10 independent runs. We report the mean and standard deviation to give an indication of the variability and robustness of the models.

5 Results

5.1 A note on reporting the results

Model performance on the laccase datasets exhibits substantial variability across different train–test splits. This phenomenon, well-documented in chemical ML research [56], arises primarily from the non-uniform coverage of chemical space: certain molecular scaffolds or functional groups are overrepresented, while others occur rarely. Generalization therefore depends not only on the model but also on how well the train and test sets overlap structurally. When they differ dramatically, even strong representations may fail to generalize. Oversampling methods such as SMOTE cannot fully resolve these issues, as they do not increase true structural diversity or compensate for representation-level limitations. More advanced strategies, such as improved representation learning, synthetic data augmentation, or transfer learning, may be required to mitigate these issues.

More broadly, the datasets used here capture only a small portion of the underlying chemical space. Sampling biases, systematic distortions, and distributional shifts may limit the extrapolative power of the models. As a result, predictions should be interpreted as conditional on the dataset's specific characteristics, rather than as broad general statements about laccase chemistry.

To more objectively evaluate model performance and its variability, we performed 10-fold nested CV on the training data for each dataset, using an 80/20 train/test split. For each split, the training data underwent 5-fold cross-validation to tune hyperparameters, with the final model evaluated on the 20% test set. This process was repeated 10 times with different random seeds to ensure the stability of the results. The final performance metrics (mean $\pm$ standard deviation) were averaged across all 10 runs.

5.2 Classical ML approaches: results

Evaluation on the test sets indicates that classical ML models are well-suited for predicting oxidation outcomes across all three datasets. For **f-tve**, the largest dataset, mean accuracies across 10 random seeds range from 0.834 to 0.864, depending on the model used (see Table 1). SVC and GradBoostC achieve the highest mean accuracies of 0.864 and 0.850. All four model families maintain a favorable trade-off between

false positives (FP) and false negatives (FN), as evidenced by F1 scores between 0.887 and 0.907, with SVC achieving the highest mean F1, followed closely by GradBoostC. The area under the receiver operating characteristic curve (AUROC) remains consistently high (0.788 – 0.882), further confirming strong class separability and robust discriminative capability.

For the **f-mth** and **bpu-lac** datasets, model performance is slightly attenuated but still demonstrates strong generalization to unseen data. On the **f-mth** dataset, mean accuracies range from 0.758 to 0.789, with corresponding F1 scores spanning 0.787 to 0.820. All four classical classifiers yield similar performance, with LogReg and RFC exhibiting slightly higher mean accuracy and F1 scores. RFC achieves the highest AUROC of 0.859, indicating superior classification confidence. For **bpu-lac**, LogReg performs strongly in terms of mean accuracy, precision and F1, whereas tree-based models have the highest AUROC values, highlighting its robustness in capturing nuanced class boundaries.

Statistical comparison using paired Wilcoxon tests indicates no significant differences between the classifiers on any of the three datasets (all $p > 0.05$). Effect sizes were uniformly small, pointing to practical equivalence in predictive performance. In such cases, model choice should follow secondary statistical criteria such as stability, robustness, and cross-dataset consistency.

Given the lack of statistically significant differences, RFC can be considered a practical choice when secondary criteria are prioritized:

- consistently competitive performance across datasets (**f-tve**, **f-mth**, **bpu-lac**), rather than excelling only on a single dataset;
- AUROC values that are consistently high relative to other models, a threshold-independent measure of class separability that is especially informative for bio/chemical tasks where class imbalance is common;
- F1 scores that remain competitive, suggesting a good balance between precision and recall at the selected operating threshold;
- comparatively low sensitivity to random seed variation, reflected by smaller standard deviations across metrics in several settings;
- generalization behavior that appears more stable in our experiments, whereas SVC and GradBoostC show near-perfect training performance but only comparable test performance, which may indicate greater susceptibility to overfitting in this setting.

Using AUROC as a primary summary metric, we ranked models within each dataset by mean AUROC (rank 1 = highest; ties assigned equal ranks) and then summed ranks across the three datasets. Under this heuristic aggregation, RFC attains the lowest summed rank (i.e., is among the top-performing models overall). When stability is summarized by the mean AUROC standard deviation across datasets, RFC exhibits the second-lowest variability (after LogReg), suggesting a favorable performance–stability trade-off.

In summary, while no classifier is statistically superior across all datasets, RFC is a reasonable default choice when prioritizing stability across random seeds and

consistent cross-dataset performance. However, alternative models remain appropriate depending on the application-specific trade-off between metrics.

Data	Method	Accuracy	Precision	Recall	F1	AUROC
**f-tve**	ChemBERTa	0.808 ± 0.056	0.868 ± 0.042	0.873 ± 0.069	0.869 ± 0.044	0.788 ± 0.094
	LogReg	0.846 ± 0.048	**0.902** ± 0.055	0.886 ± 0.050	0.893 ± 0.042	0.878 ± 0.052
	SVC	**0.864** ± 0.040	0.897 ± 0.052	0.920 ± 0.053	**0.907** ± 0.037	**0.882** ± 0.046
	RFC	0.834 ± 0.064	0.872 ± 0.061	0.903 ± 0.056	0.887 ± 0.054	0.856 ± 0.074
	GradBoostC	0.850 ± 0.051	0.880 ± 0.059	**0.920** ± 0.040	0.898 ± 0.043	0.870 ± 0.078
	Ensemble	0.872 ± 0.041	0.895 ± 0.051	0.934 ± 0.036	0.914 ± 0.035	0.893 ± 0.053
**f-mth**	ChemBERTa	0.760 ± 0.053	0.756 ± 0.054	0.818 ± 0.080	0.782 ± 0.041	0.840 ± 0.058
	LogReg	0.784 ± 0.051	**0.787** ± 0.063	0.823 ± 0.082	0.801 ± 0.039	0.834 ± 0.060
	SVC	0.766 ± 0.065	0.758 ± 0.073	0.842 ± 0.112	0.791 ± 0.047	0.855 ± 0.071
	RFC	**0.789** ± 0.039	0.751 ± 0.050	**0.911** ± 0.078	**0.820** ± 0.029	**0.859** ± 0.046
	GradBoostC	0.758 ± 0.043	0.733 ± 0.057	0.857 ± 0.079	0.787 ± 0.040	0.849 ± 0.047
	Ensemble	0.772 ± 0.064	0.775 ± 0.066	0.837 ± 0.094	0.799 ± 0.049	0.857 ± 0.055
**bpu-lac**	ChemBERTa	0.779 ± 0.076	0.789 ± 0.094	0.796 ± 0.088	0.789 ± 0.079	0.841 ± 0.070
	LogReg	**0.800** ± 0.062	**0.798** ± 0.072	**0.833** ± 0.074	**0.813** ± 0.058	0.844 ± 0.057
	SVC	0.782 ± 0.091	0.768 ± 0.070	0.844 ± 0.142	0.798 ± 0.089	0.839 ± 0.092
	RFC	0.791 ± 0.073	0.771 ± 0.081	0.854 ± 0.084	0.809 ± 0.073	0.857 ± 0.067
	GradBoostC	0.791 ± 0.066	0.771 ± 0.072	0.854 ± 0.090	0.808 ± 0.070	**0.866** ± 0.076
	Ensemble	0.812 ± 0.069	0.798 ± 0.087	0.851 ± 0.107	0.821 ± 0.080	0.875 ± 0.053

Table 1 Performance comparison across all approaches. Best per metric model (bold) is reported, excluding the Ensemble

5.3 ChemBERTa fine-tuning: results

The fine-tuned ChemBERTa model achieves performance comparable to the four classical ML methods, albeit with slightly reduced discriminative power on the **f-tve** and **f-mth**, and modestly lower accuracy across all three datasets. Nonetheless, the model maintains strong overall performance, with mean accuracies exceeding 0.760 for all three datasets. On the **f-tve** dataset, ChemBERTa achieves a mean F1 score of 0.869, reflecting a good precision-recall balance, though F1 scores are lower for the smaller and more structurally limited **f-mth** and **bpu-lac** datasets. The AUROC values for ChemBERTa span 0.788 to 0.841 across different datasets with the highest value for **bpu-lac** and slightly diminished values observed on the other two datasets, reflecting reduced, when compared to the classical classification approaches, but still meaningful class separability.

A comprehensive comparison of the performance metrics across all five approaches is provided in Table 1.

5.4 Results interpretation based on RFC feature scores

To gain insight into the decision-making process of classical ML algorithms, we analyze feature importance scores from the `RandomForestClassifier()`. RFC feature importances are based on the reduction in Gini impurity $\mathcal{G}_|$ calculated for each feature $j = 1, \ldots, p$ after it has been split at value τ into two child nodes, containing S_g^L and

S_g^R samples:

$$\Delta\mathcal{G}_j = \mathcal{G}_j(S_g) - \left(\frac{|S_g^L|}{|S_g|}\mathcal{G}_j^L + \frac{|S_g^R|}{|S_g|}\mathcal{G}_j^R\right),$$

which are then averaged across all features $\Delta\mathcal{G} = \frac{1}{(p-1)}\sum_{j=1}^{(p-1)}\Delta\mathcal{G}_j$ and normalized to sum to 1. While RFC feature importances are easy to compute, they can be biased towards high-cardinality features and do not capture interactions between features.

A comparative analysis across multiple random splits and datasets reveals recurring patterns, in which the RFC repeatedly returns to the same families of molecular descriptors when explaining substrate activity. Across all three datasets, the most influential features cluster around

(i) constitutional descriptors that quantify unsaturation and reactivity, such as the number of multiple (double/triple) bonds and the presence of *sp*-hybridized carbons,
(ii) global molecular properties linked to aromaticity and stability, including measures of total unsaturation,
(iii) functional-group and hybridization counts, for example aliphatic sp^2 carbons that shape steric and electronic behaviour, and
(iv) ring-system complexity, captured by detour-based indices that reflect cyclization and conformational rigidity.

This core set is complemented by two broader descriptor families that also recur across datasets: P_VSA-like descriptors, which encode surface-area and volume contributions modulated by electronic properties (e.g., ionization potential), and topological indices that summarize molecular size, branching, and shape asymmetry (e.g., all-path Wiener index, Petitjean index, and intrinsic-state pseudoconnectivity). Taken together, these signals suggest that the models primarily discriminate substrates through a combination of reactivity-related unsaturation, steric constraints, and topology-driven accessibility.

Within this shared foundation, each dataset has characteristic signatures. For **f-tve**, feature importance consistently highlights the number of rotatable bonds, pointing to molecular flexibility as a key differentiator for this enzyme set. For **f-mth**, the models place more weight on descriptors tied to polarity and transport, including the number of heteroatoms and the Moriguchi logP index, alongside the count of non-aromatic conjugated $C(sp^2)$ atoms, which likely captures how conjugation and rigidity influence reactivity. In contrast, **bpu-lac** shows a stronger reliance on structural/topological descriptors (e.g., Narumi harmonic index and lopping centric index), indicating that two-dimensional connectivity and graph structure carry relatively more predictive power in the bacterial dataset.

These patterns also reveal a biologically intuitive relationship between enzyme groups. The two fungal datasets (**f-tve** and **f-mth**) display more similar importance profiles to one another than to the bacterial set (**bpu-lac**), implying closer shared determinants of substrate recognition among fungal laccases. In particular, the stronger prominence of P_VSA-like descriptors for the fungal datasets suggests that

surface area, volume, and related steric–electronic properties of the substrates are especially informative for oxidation-catalyzing capability in these enzymes.

These findings align with known biochemical mechanisms and enzyme-substrate interaction principles. Unsaturation, ring structures, and aromaticity features influence electron delocalization, redox potential, and substrate binding affinity, all of which are central to laccase-mediated oxidation reactions [82]. Topological and surface area descriptors, capture steric accessibility, molecular shape, and electrostatic surface characteristics, which are crucial for determining how well a substrate fits into the active site of the enzyme – especially for fungal laccases, which often act on bulkier, aromatic, or polymeric compounds like lignin [13, 83]. Finally, fungal laccases tend to oxidize a broader range of substrates and are more influenced by surface-interacting descriptors while bacterial laccases may depend more on compact structural features and connectivity, reflecting tighter or more selective binding pockets [83].

5.5 Comparison of RFC-derived feature scores with the Markov blanket

To complement the feature importance analysis, we explore probabilistically grounded feature selection via Bayesian Networks (BNs), trained over the set of molecular descriptors.

A BN is a directed acyclic graph (DAG), characterized by a pair (G, Θ), where each node G represents a random variable - the (binned) descriptors $\mathbf{x}_{j'}, j' = 1, \ldots, p$, including the target variable y, and directed edges indicate dependency; Θ denotes the set of conditional probability distributions (CPDs) for each $\mathbf{x}_{j'}$, given its parents in the graph $Pa(\mathbf{x}_{j'})$.

The BN encodes the joint probability distributions of all variables as

$$P(\mathbf{x}_1, \ldots, \mathbf{x}_{j'}) = \prod_{j'}^{p} P(\mathbf{x}_{j'} \mid Pa(\mathbf{x}_{j'})).$$

A Markov blanket of a target variable y is the minimal set of features that renders y conditionally independent of all other variables. This subset captures the most relevant information for prediction and serves as a theoretically sound basis for feature selection.

To prepare continuous variables for structure learning in `pgmpy`, [84], which requires discrete inputs, we apply the minimum description length principle (MDLP) discretization algorithm [85]. Unlike fixed-width or quantile binning, MDLP automatically adapts the number and position of bins to the complexity of the underlying distribution. It finds a cut point t that maximizes the information gain between a continuous variable X_j and a discrete target Y:

$$\mathrm{IG}(t) = H(Y) - \left(\frac{n_L}{n} H(Y_L) + \frac{n_R}{n} H(Y_R)\right),$$

where $H()$ is Shannon entropy, n_L, n_R and Y_L, Y_R are respectively the number of observations and label distributions after splitting at t. This point is accepted only if

exceeds the MDLP threshold

$$\mathrm{IG}(t) > \frac{\log_2(n-1)}{n} + \frac{\Delta}{n},$$

where Δ penalizes the increased model complexity. This leads to clearer causal dependencies, more accurate Markov blankets, and more interpretable networks—especially with small datasets (as in our case), where over-fragmentation must be avoided.

To learn the probabilistic structure, we fit a discrete BN using the MDLP-discretized training set. BN learning involves structure learning (recovering the DAG that encodes conditional dependencies) and parameter learning, which estimates the associated conditional probability tables (CPTs).

For structure learning, we use Hill-Climb Search, a score-based algorithm that iteratively adds, deletes, or reverses edges to maximize a model selection criterion. We employ the Bayesian Information Criterion (BIC), which balances goodness-of-fit with structural parsimony and performs well on small datasets by penalizing overly complex graphs.

The CPTs are learned via Maximum Likelihood Estimation (MLE), which computes conditional probabilities directly from observed frequencies in the discretized data—an efficient and statistically appropriate choice for discrete variables and limited sample sizes. After fitting the full network, we extract the Markov Blanket of the target variable (Oxd).

This provides a principled feature-selection mechanism, isolating the minimal set of variables that uniquely determine the predictive behavior of the target and guiding all downstream modeling and interpretation.

Comparison with the RFC features reveals substantial overlap, with some key differences. The Markov blankets often emphasize constitutional descriptors (e.g., average molecular weight, mean van der Waals volume, first ionization potential), while P_VSA-like descriptors appear less frequently than in the RFC analysis. Atom-specific counts (e.g., oxygen or carbon) are common in the Markov blanket but rarely appear among the top RFC features.

These differences highlight the complementary nature of the two methods: RFC scores are influenced by model-specific dynamics, while the Markov blanket reflects the intrinsic statistical dependencies in the data.

Figure 2 shows the RFC features with the importance score exceeding 0.01 and the Markov blanket for the **bpu-lac** data.

5.6 Attention patterns and interpretability in ChemBERTa

One of the compelling aspects of BERT-based architectures is their ability to offer insights into the internal patterns learned during training through the attention mechanisms in each layer and head. These attention dynamics can be explored using the `head_view` module from the BERTViz library [86], or by directly visualizing the self-attention weight matrices (see Figure S2 and Figure S3) across all attention heads.

While these visualizations provide useful information, interpreting the complex attention patterns, particularly given the model's size and depth, remains a challenge for humans.

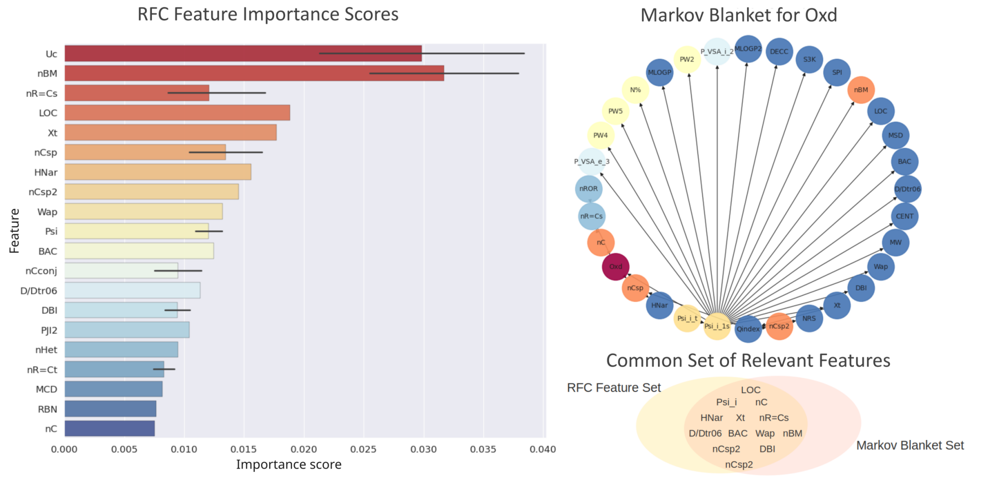

Figure 2 RFC feature importances and Markov blanket for **bpu-lac**(7890). Error bars for the feature importance scores are displayed whenever a descriptor was selected as a splitting feature in more than one of the 10 random train/test splits. Descriptors that were used in only a single split do not exhibit variance across splits and therefore appear without error bars.

To obtain a better explanation of the model's predictions, we turn to layer integrated gradients (LIGs), computed via the captum library. Layer Integrated Gradients (LIG) provide token-level attributions based on the output of the final classification layer, highlighting which tokens, representing substructures in the molecular graph, influence the prediction both in terms of direction (positive or negative sign) and strength (magnitude). In Figure 3, substructures contributing positively to the prediction of the oxidation are highlighted in blue, those contributing negatively – in red. Structures with minimal, or neutral, influence are marked in yellow[6].

We also map SMILES-based attributions to the corresponding substructures in the molecular graph. While not perfectly precise, this mapping offers a valuable visual aid for identifying which chemical motifs the model considers influential in determining whether a substrate can be oxidized by the given laccase. The procedure for mapping token-level attributions to molecular graphs is outlined in Figure 4 and is rigorously documented in the repository.

A closer inspection of the LIG attributions tells a fairly coherent chemical story, which is largely consistent across datasets. Fragments typically known to facilitate oxidation, such as phenolic OH groups, electron-rich aromatic rings, methoxy substituents, and several heteroaromatic motifs, are repeatedly assigned positive contributions. This is particularly clear in **f-mth**, where multiple examples show strong positive attribution around motifs like $c1cc(OC)ccc1O$, aligning with the established role of phenols as common laccase substrates. Similar trends appear in **bpu-lac**, where aromatic ether patterns such as $COC1 = CC$ and $c1cc(OC)$ are often highlighted positively, again pointing to electron-donating substitution on aromatic systems as a recurring signal.

[6]We use a custom visualization approach in place of the `captum`'s `visualization.visualize_text()` function.

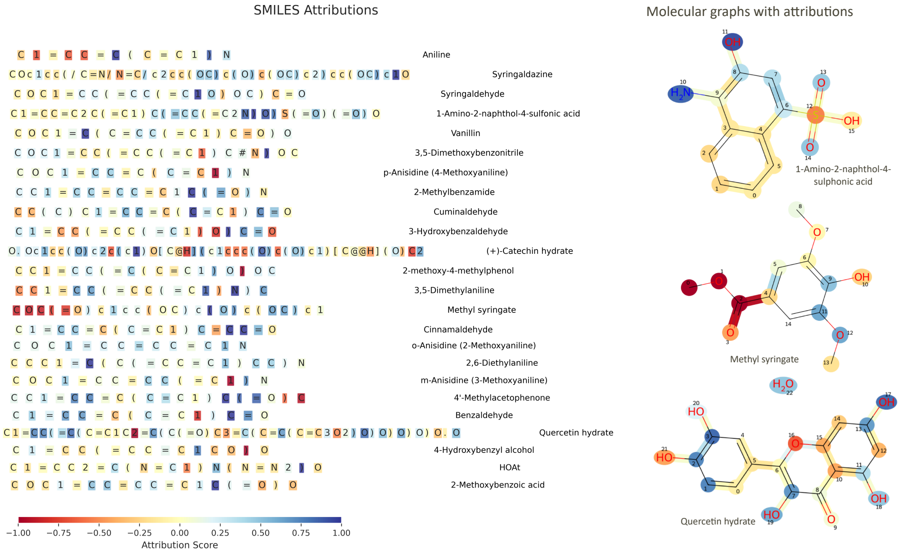

Figure 3 Layer integrated gradient attributions for substrate substructures marked in SMILES and in the molecular graph, **bpu-lac**(7)

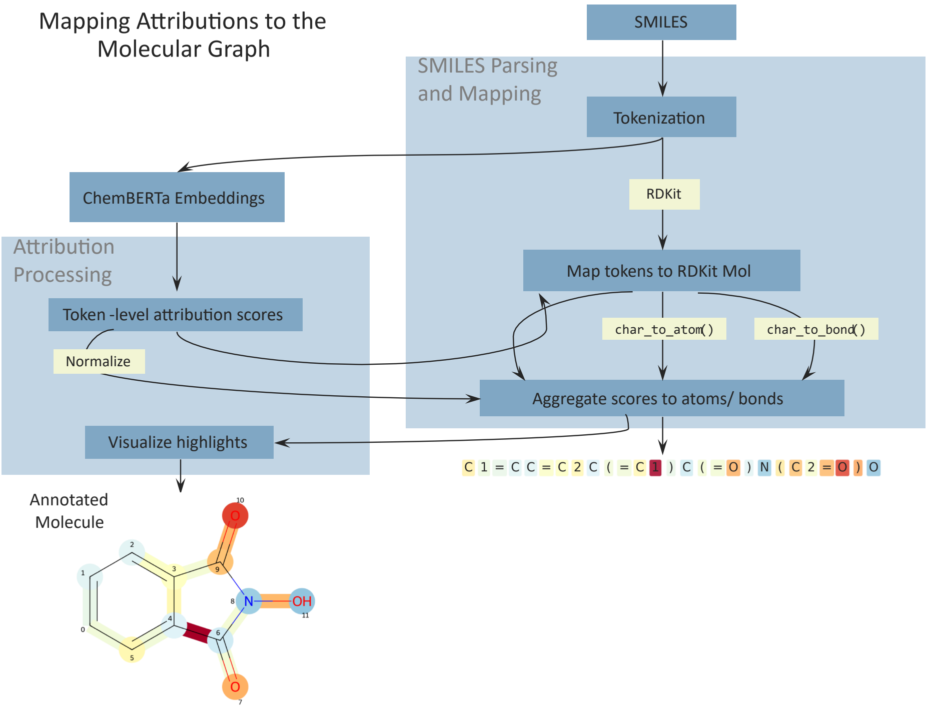

Figure 4 Workflow of mapping token-level attributions to the molecular graph

By contrast, substituents that reduce electron density or otherwise impede access to reactive sites tend to pull predictions in the opposite direction. Electron-withdrawing groups, including nitro (NO_2), halogens, and carboxyl functionalities, are frequently associated with negative contributions, while bulky saturated moieties and non-conjugated aliphatic chains tend to register as neutral or weakly informative. In both **f-tve** and **bpu-lac**, fragments such as sulfonamides (SO_2N) and trifluoromethyl groups (CF_3) are commonly mildly negative or near-neutral, which is consistent with the idea that strong electron withdrawal and steric burden can hinder oxidation rather than promote it.

The behavior of nitrogen-containing aromatic heterocycles is more nuanced, and the attributions reflect that nuance rather than forcing a single rule. Across **f-tve** and **bpu-lac**, heterocycles such as indoles, imidazoles, and triazoles can swing either way: some ring nitrogens, for instance in patterns like $n1cccn1$, appear positively contributing, whereas others become neutral or negative when embedded in more substituted or sterically constrained environments. This mixed behaviour suggests the model is sensitive to where nitrogen sits in the ring and what surrounds it (substitution pattern, adjacent electron-withdrawing or donating groups, and local steric context), which is chemically plausible given how these factors modulate ring electronics and accessibility.

Explicit stereochemical markers in SMILES (@, @@, $[C@H]$) tend to carry neutral or mildly negative attribution. This suggests that, for the oxidation outcomes captured here, stereochemistry is not a dominant determinant of the model's decision, which is consistent with the working assumption that chirality often has a smaller effect on redox propensity than electronic activation and functional-group context.

Finally, some small irregularities in attribution are best interpreted as artefacts of the representation rather than true chemistry. Because the explanations are computed over SMILES tokens, junction symbols and syntactic elements (parentheses, ring closures such as 1, 2, and bond symbols like =) can accumulate "noisy" or mixed contributions, for example around frequent fragments like $C(=O)$ or $c1cc$. This is a known limitation of string-based parsing and reinforces the benefit of projecting attributions back onto the molecular graph, where contributions can be aggregated over chemically meaningful substructures instead of SMILES syntax.

In summary, all three laccases demonstrate positive attribution for electron-rich aromatics and linear conjugated systems, negative attribution for electron-withdrawing groups and ambiguous attribution for N-heterocycles. The bacterial laccase appears more selective and functionally focused, likely reflecting a narrower substrate range or tighter active site constraints, while the two fungal laccases show broader reactivity, suggesting a broader substrate scope or less stringent structural specificity.

6 Discussion

6.1 Data augmentation

The obtained results suggest potential for improving model performance, stability, and generalization. Data augmentation has been widely recognized as an effective strategy when the available data is limited or imbalanced [87–92].

We explore Gaussian noise (GN) injection, a simple and effective method used in molecular modeling with continuous descriptors [87]. Noise is added to the training data prior to preprocessing: for each descriptor $j = 1, \ldots, p$, the perturbed feature is defined as $\mathbf{x}_{\cdot j} + \xi_j$, where $\xi_j \sim \mathcal{N}(0, \eta\sigma_j)$ and η represents the noise intensity. We experiment with the noise levels $\eta = \{0.1, 0.2\}$. Ordinal descriptors are handled by adding noise, rounding to the nearest integer, and clipping values within the observed range. Labels remain unchanged.

GN injection is appealing due to its simplicity and ease of implementation for numeric variables. Furthermore, it does not impose strong distributional assumptions on the data. However, in mixed-type datasets like ours, where both continuous and discrete descriptors are present, the method must be adapted carefully to avoid distortions of the original data distribution.

Retraining all four classifiers on the augmented data with $\eta = 0.1$ shows negligible improvement over the baseline, while $\eta = 0.2$ significantly reduces performance (see Table ST2). Synthetic data generation methods like CTGAN (Conditional Tabular Generative Adversarial Network) or nearest-neighbor face challenges with sparse chemical datasets due to limited feature space coverage.

For SMILES data, where multiple valid representations of a molecule exist, we augment the datasets by generating all valid SMILES strings using the `RDKit` package, retaining the corresponding labels. This strategy allows to obtain approximately 2000, 1500 and 1400 training examples for the **f-tve**, **f-tve** and **bpu-lac**.

SMILES augmentation improves ChemBERTa performance, showing stable increases across all metrics and data splits (see Table ST3), suggesting its beneficial impact.

6.2 Ensemble learning and stacked generalization

We explore an ensemble learning strategy based on averaged probability voting across the four classical classifiers, which outperform the transformer-based model. The goal is to improve accuracy, generalization, stability, and reduce overfitting. For each instance, predicted probabilities of the positive class are averaged across the models, and a decision threshold of 0.5 is applied to derive the final prediction. The performance comparison of this ensemble model is shown in Figure 1. While the ensemble approach provides modest improvements, with higher mean performance metrics and lower standard deviations for **f-tve** and **f-mth**, it shows no gain for the **bpu-lac** dataset.

Analyzing ChemBERTa's false negatives (FN) via mapped token-level attributions reveals common patterns, such as electron-withdrawing groups affecting oxidizable sites, substituted aromatic amines with deactivating groups, and phenolic compounds

with competing functional groups. These FN cases suggest that the model associates certain functional groups (e.g., NH_2, phenolic OH) with "non-reactivity" in specific environments, likely due to electron-withdrawing substituents. This likely points to the model learning overly simplistic rules, e.g. "aromatic amines with electron-withdrawing substituents are not oxidizable".

Laccases are powerful oxidizing enzymes, capable of overcoming moderate electronic deactivation, so the model could benefit from better representation of edge cases where laccases oxidize electronically deactivated but still chemically accessible substrates.

As mentioned above, descriptor-based and SMILES-based representations can be seen as complementary for ML modeling: molecular descriptors capture specific molecular properties by calculating physicochemical properties, encoding topological indices and $3D$ structural information, while SMILES contain implicit structural information in sequential format, preserving exact connectivity patterns and stereochemistry and maintain complete molecular graph topology. To leverage both, we adopt a stacked generalization approach, hypothesizing that different model architectures capture complementary data patterns, and aggregating their predictions could enhance performance.

For stacked generalization, we use a dedicated validation set to train the meta-model. The splits (train/validation/test) are as follows: 180/49/21 for **f-tve**, 136/37/17 for **f-mth**, and 122/33/15 for **bpu-lac**.

The stacking procedure consists of the following steps:

1. Train the four classical classifiers and ChemBERTa on their respective representations.
2. Compute prediction probabilities for all models on the validation set.
3. For each data point $v = 1, \ldots, n_V$ in the validation set of size n_V and model $k = 1, \ldots, 5$, a confidence score is computed as $s_{v,k} = 2 \cdot (\hat{P}_{v,k}^{\text{true}} - 0.5)$, where $\hat{P}_{v,k}^{\text{true}}$ is the estimated probability of the true class.
4. Embed the descriptor feature space into $2D$ using Isomap to visualize "regions of confidence", illustrated via Voronoi tessellation (Figure 5).
5. To classify a new sample, project into the same $2D$ Isomap space. A KDTree meta-model retrieves the nearest validation point and assigns the class predicted by the base model with the highest local confidence.

An overview of the stacked generalization strategy is provided in Figure 5 while comparison with the classical classifiers and ChemBERTa can be found in Table 2. Despite its intuitive appeal, stacked generalization does not yield significant improvement over standalone classical classifiers.

While marginal gains are observed for **f-tve** and **f-mth**, these often come with higher variance across splits, and performance is lower than that of the classical classifiers on **bpu-lac**. Several factors likely contribute to this. Firstly, feature space distortion by Isomap: the $2D$ projection may inadequately capture the true structure of the high-dimensional feature space, especially in the presence of complex nonlinear interactions. Secondly, confidence score transformation, since shifting probabilities

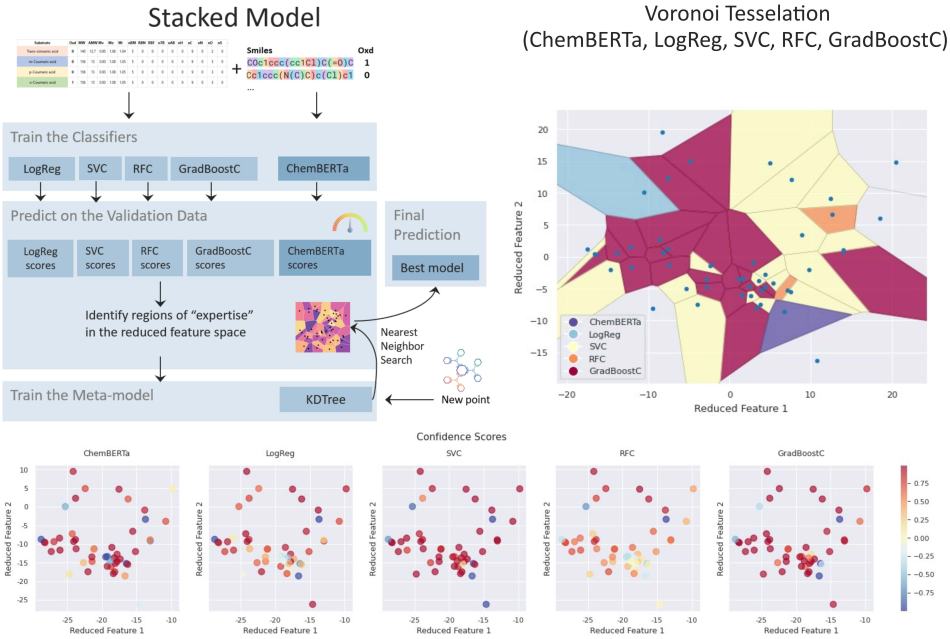

Figure 5 Stacked model architecture and Voronoi diagram for f-tve

around zero may overemphasize uncertainty near the decision boundary and misalign confidence with actual predictive power. Lastly, meta-model limitations, as KDTree assumes that local proximity correlates with predictive success, which may not hold consistently across diverse regions of the feature space. Small validation sets further increase the risk of overfitting.

Taken together, these results suggest that, as a standalone approach, RFC offers a strong practical balance of simplicity, interpretability, robustness across splits, and reliable generalization in this setting. Where maximizing predictive performance is the primary objective and additional time and computational cost are acceptable (with reduced interpretability), the ensemble provides a viable alternative.

Conclusions

This study evaluates ML approaches for predicting laccase-mediated oxidation of aromatic substrates, comparing classical classifiers using molecular descriptors with a fine-tuned ChemBERTa transformer model using SMILES representations, benchmarked across three novel datasets specific to different laccase enzymes.

Summary of findings

Both classical and transformer-based approaches effectively predict laccase-substrate reactivity. All four classical ML models (LogReg, SVC, RFC, and GradBoostC) achieve robust predictive performance across the three datasets, with high accuracy (> 0.84), F1(> 0.88) and AUROC (> 0.85) for the largest **f-tve** dataset and slightly

Data	Method	Accuracy	Precision	Recall	F1	AUROC
**f-tve**	ChemBERTa	0.781 ± 0.093	0.846 ± 0.086	0.848 ± 0.114	0.841 ± 0.075	0.809 ± 0.135
	LogReg	0.824 ± 0.071	0.872 ± 0.061	0.878 ± 0.088	0.873 ± 0.061	0.866 ± 0.088
	SVC	0.843 ± 0.080	**0.896** ± 0.059	0.880 ± 0.081	0.887 ± 0.062	0.860 ± 0.076
	RFC	0.829 ± 0.077	0.864 ± 0.044	0.899 ± 0.105	0.878 ± 0.061	0.808 ± 0.177
	GradBoostC	**0.843** ± 0.102	0.887 ± 0.078	0.894 ± 0.078	0.889 ± 0.071	0.818 ± 0.170
	Stacked*	0.843 ± 0.077	0.875 ± 0.053	**0.906** ± 0.071	**0.889** ± 0.059	**0.869** ± 0.127
**f-mth**	ChemBERTa	0.753 ± 0.105	0.782 ± 0.125	0.788 ± 0.130	0.776 ± 0.094	0.832 ± 0.092
	LogReg	0.765 ± 0.070	0.788 ± 0.092	0.791 ± 0.071	0.786 ± 0.066	0.810 ± 0.103
	SVC	0.788 ± 0.099	**0.815** ± 0.105	0.797 ± 0.108	0.803 ± 0.095	0.825 ± 0.107
	RFC	**0.800** ± 0.096	0.782 ± 0.094	**0.883** ± 0.081	**0.828** ± 0.082	**0.860** ± 0.070
	GradBoostC	0.783 ± 0.078	0.788 ± 0.066	0.846 ± 0.107	0.811 ± 0.065	0.840 ± 0.070
	Stacked*	0.732 ± 0.054	0.773 ± 0.124	0.787 ± 0.097	0.768 ± 0.061	0.829 ± 0.063
**bpu-lac**	ChemBERTa	0.753 ± 0.095	0.773 ± 0.135	0.801 ± 0.150	0.772 ± 0.096	0.851 ± 0.059
	LogReg	0.767 ± 0.120	0.843 ± 0.124	0.745 ± 0.255	0.745 ± 0.197	0.844 ± 0.100
	SVC	0.800 ± 0.094	0.843 ± 0.141	0.775 ± 0.176	0.791 ± 0.126	0.888 ± 0.086
	RFC	0.787 ± 0.063	**0.879** ± 0.125	0.772 ± 0.209	0.791 ± 0.157	0.888 ± 0.094
	GradBoostC	**0.833** ± 0.068	0.853 ± 0.116	**0.859** ± 0.090	**0.847** ± 0.061	**0.891** ± 0.089
	Stacked*	0.787 ± 0.102	0.839 ± 0.119	0.767 ± 0.205	0.780 ± 0.132	0.863 ± 0.103

Table 2 Performance of the stacked model compared with baseline approaches (using train/validation/test splits). Best values are shown in bold; in the case of ties, the model with the lower standard deviation is preferred

lower but reliable performance for the **f-mth** and **bpu-lac**. ChemBERTa performed marginally worse on this prediction task. Although no model is statistically superior across all laccases, RFC is a practical standalone choice that balances simplicity, interpretability, robustness across splits, and generalization. If maximum performance is the priority and additional compute is acceptable, the ensemble is a viable, though less interpretable, alternative.

Feature importance analysis highlighted molecular descriptors related to unsaturation, aromaticity, electronic properties, and topology as key to predicting oxidation outcomes. Notably, multiple bonds, *sp*-hybridized carbons, functional group counts, and ring system descriptors were identified as important features.

ChemBERTa's layer-integrated gradient analysis identified meaningful chemical substructures that contribute positively (aromatic rings, conjugated systems) or negatively (aliphatic side chains, isolated ketones) to oxidation prediction, aligning with established chemical principles.

Limitations

Despite the promising results, several limitations remain that should be considered. First, the size and diversity of the datasets pose significant constraints. With only 172 to 250 compounds per enzyme, and datasets predominantly composed of benzenoid aromatics, the models may fail to capture the full range of chemical diversity necessary to generalize to broader chemical spaces. Additionally, the performance of the models varied across different train-test splits, underscoring the challenge of working with limited and potentially non-representative data in chemical machine learning.

While the models demonstrate good accuracy in predicting binary oxidation outcomes, they fall short of providing deeper insights into reaction kinetics, product

distributions, and the underlying mechanisms of oxidation. These aspects are critical for developing a more comprehensive understanding of the oxidation process.

Finally, current interpretability methods often require substantial expert knowledge to translate the computational results into actionable chemical insights, limiting their practical use for non-expert researchers or in applied settings.

Future directions

Building on this foundation, several promising research directions can be taken into consideration.

Expanding the datasets with additional substrates and enzymes to improve model generalization and enable cross-enzyme prediction capabilities:

1. Developing multi-task models that can simultaneously predict oxidation outcomes across multiple laccases, potentially revealing shared and enzyme-specific reactivity patterns.
2. Concatenating molecular descriptors with SMILES-derived embeddings and possibly other representations prior to feeding them into a suitable classifier.
3. Exploring more advanced model architectures, such as graph neural networks that can directly learn from molecular structures rather than pre-computed descriptors or linearized SMILES representations.
4. Integrating these predictive models into computational workflows for enzyme engineering or substrate design, enabling the rational development of novel laccase applications.

In conclusion, this work demonstrates the feasibility and utility of machine learning approaches for predicting laccase-mediated oxidation outcomes. The developed models and methodologies provide a valuable framework for more efficient enzyme-substrate matching in various biotechnological applications, reducing the reliance on costly and time-consuming experimental screening. As datasets grow and modeling techniques advance, these approaches hold significant promise for accelerating the development of green oxidation catalysts across multiple industries.

Funding

NA

Conflict of interest

There are no conflicts to declare.

Ethics approval and consent to participate

NA

Consent for publication

All authors have read and approved the final version of the manuscript. We confirm that this work is original, has not been published elsewhere, and is not under consideration by any other journal.

Data availability

The datasets generated and/or analyzed in this study are archived on Zenodo under 10.5281/zenodo.15728331. In accordance with institutional data-governance requirements at Empa (Department 302), the files are provided via a controlled-download procedure that records intended use. To obtain access, submit a request to the data custodian (M. Schubert) including: (i) name and institutional affiliation, (ii) contact email, (iii) a brief description of the intended use (e.g., reproducibility check, method development, benchmarking), and (iv) whether the use is academic/non-commercial or commercial. Requests are approved without cost for academic and other non-commercial research purposes. Approved users may download the data for research use, may redistribute only derived results (e.g., trained models, aggregate statistics), and should not redistribute the raw dataset files outside their research group without permission. Any publications using the data should cite the Zenodo record 10.5281/zenodo.15728331 and this article. Commercial use or redistribution requires prior written authorization from Empa.

Code availability

The accompanying code and further implementation details are available in the GitHub repository: https://github.com/empa-woodtec/laccase-oxd.

Author contributions

Yulia Kulagina: conceptualization, data curation, formal analysis, validation, visualization, writing - original draft preparation, writing – review and editing. Christian Goldhahn: investigation, writing – review and editing. Ramon Weisshaupt: investigation, writing – review and editing. Mark Schubert: data curation, supervision, resources.

Acknowledgements

We used OpenAI's ChatGPT (GPT-5) for editorial assistance and to improve the precision of mathematical expressions under the authors' direction.

Supplementary material

Figures and Tables

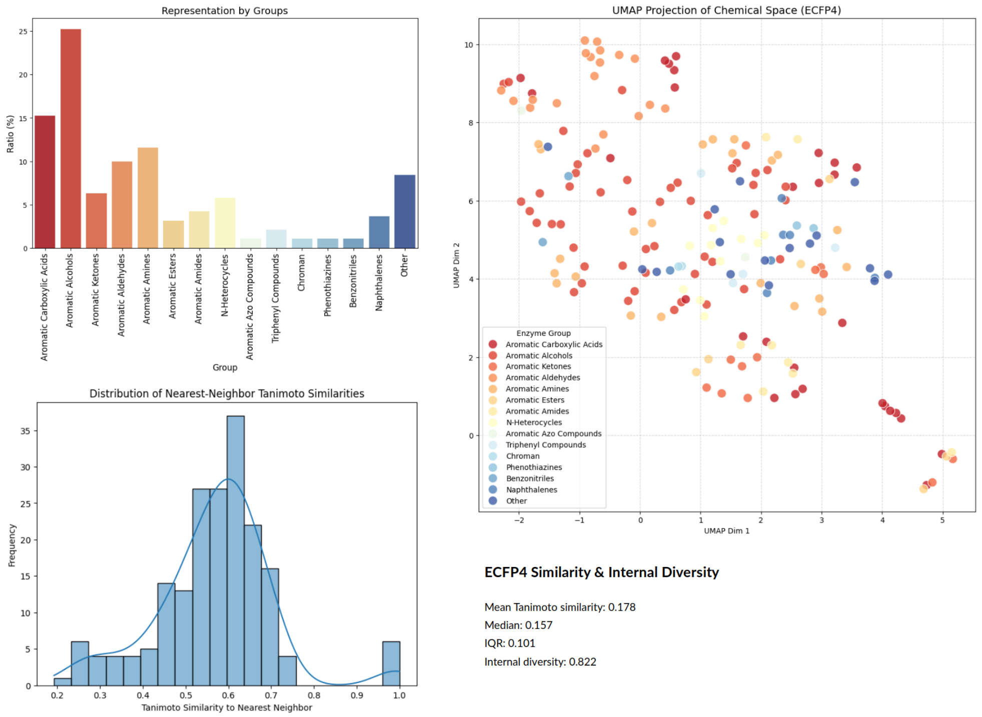

Figure S1 Dataset diversity analysis (**f-mth**)

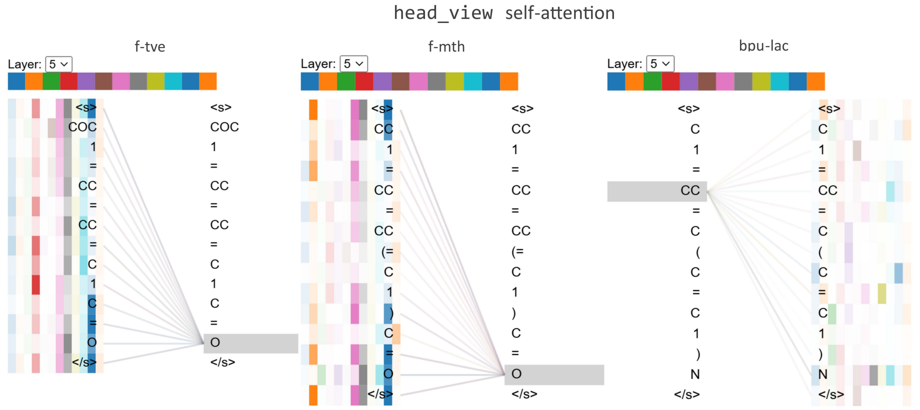

Figure S2 head_view for **f-tve**(9876), **f-mth**(876), and **bpu-lac**(76) datasets for the 6th layer

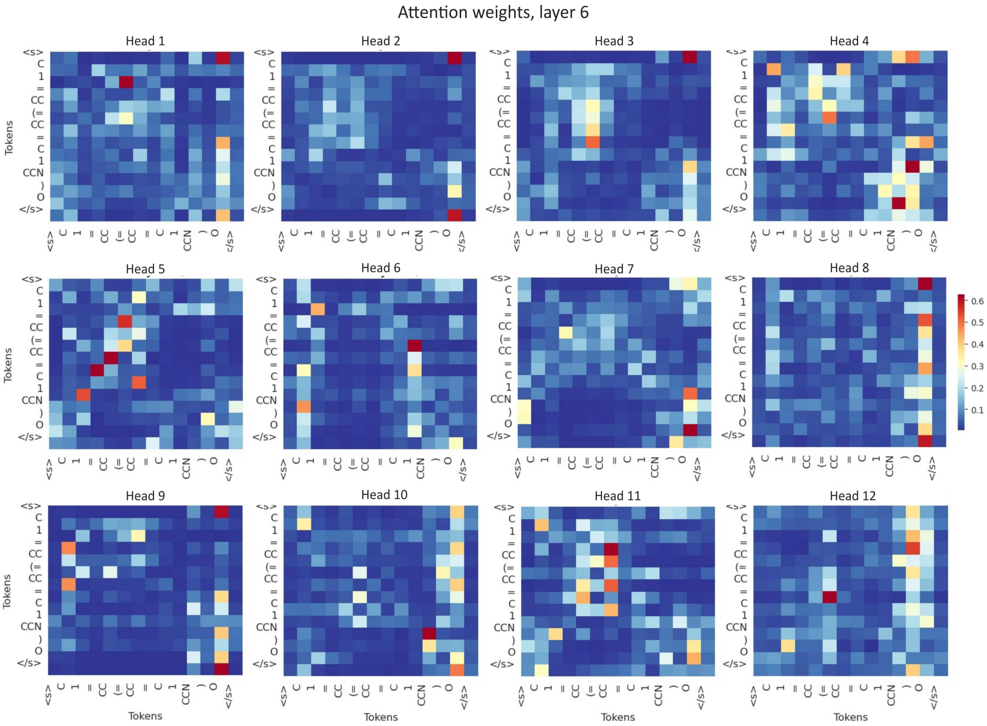

Figure S3 Self-attention weights for **bpu-lac**(76), 6-th layer

Data	Method	Strategy 1 Accuracy	Strategy 1 Precision	Strategy 1 Recall	Strategy 1 F1	Strategy 1 AUROC	Strategy 2 Accuracy	Strategy 2 Precision	Strategy 2 Recall	Strategy 2 F1	Strategy 2 AUROC
**f-tve**	**LogReg**	**0.846** $\pm$ 0.048	**0.902** $\pm$ 0.055	**0.886** $\pm$ 0.050	**0.893** $\pm$ 0.042	**0.878** $\pm$ 0.052	0.816 $\pm$ 0.074	0.882 $\pm$ 0.069	0.864 $\pm$ 0.060	0.872 $\pm$ 0.058	0.849 $\pm$ 0.080
	**SVC**	**0.864** $\pm$ 0.040	0.897 $\pm$ 0.052	**0.920** $\pm$ 0.053	**0.907** $\pm$ 0.037	0.882 $\pm$ 0.046	0.864 $\pm$ 0.062	**0.911** $\pm$ 0.047	0.897 $\pm$ 0.069	0.904 $\pm$ 0.054	**0.891** $\pm$ 0.063
	**RFC**	**0.834** $\pm$ 0.064	0.872 $\pm$ 0.061	**0.903** $\pm$ 0.056	**0.887** $\pm$ 0.054	**0.856** $\pm$ 0.074	0.832 $\pm$ 0.053	**0.877** $\pm$ 0.058	0.896 $\pm$ 0.039	0.886 $\pm$ 0.044	0.847 $\pm$ 0.089
	**GradBoostC**	0.850 $\pm$ 0.051	0.880 $\pm$ 0.059	0.920 $\pm$ 0.040	0.898 $\pm$ 0.043	0.870 $\pm$ 0.078	**0.874** $\pm$ 0.057	**0.897** $\pm$ 0.060	**0.933** $\pm$ 0.043	**0.914** $\pm$ 0.047	**0.885** $\pm$ 0.080
**f-mth**	**LogReg**	**0.784** $\pm$ 0.051	**0.787** $\pm$ 0.063	0.823 $\pm$ 0.082	**0.801** $\pm$ 0.039	**0.834** $\pm$ 0.060	0.758 $\pm$ 0.071	0.737 $\pm$ 0.066	**0.859** $\pm$ 0.089	0.789 $\pm$ 0.049	0.817 $\pm$ 0.066
	**SVC**	0.766 $\pm$ 0.065	0.758 $\pm$ 0.073	0.842 $\pm$ 0.112	0.791 $\pm$ 0.047	**0.855** $\pm$ 0.071	**0.792** $\pm$ 0.050	**0.780** $\pm$ 0.035	**0.849** $\pm$ 0.091	**0.810** $\pm$ 0.043	0.845 $\pm$ 0.047
	**RFC**	**0.789** $\pm$ 0.039	**0.751** $\pm$ 0.050	**0.911** $\pm$ 0.078	**0.820** $\pm$ 0.029	**0.859** $\pm$ 0.046	0.784 $\pm$ 0.035	0.748 $\pm$ 0.054	0.899 $\pm$ 0.076	0.813 $\pm$ 0.028	0.858 $\pm$ 0.056
	**GradBoostC**	0.758 $\pm$ 0.043	0.733 $\pm$ 0.057	0.857 $\pm$ 0.079	0.787 $\pm$ 0.040	**0.849** $\pm$ 0.047	**0.766** $\pm$ 0.059	**0.746** $\pm$ 0.079	**0.859** $\pm$ 0.066	**0.795** $\pm$ 0.040	0.845 $\pm$ 0.061
**bpu-lac**	**LogReg**	0.800 $\pm$ 0.062	**0.798** $\pm$ 0.072	0.833 $\pm$ 0.074	0.813 $\pm$ 0.058	0.844 $\pm$ 0.057	**0.806** $\pm$ 0.046	0.788 $\pm$ 0.083	**0.862** $\pm$ 0.089	**0.819** $\pm$ 0.058	**0.853** $\pm$ 0.069
	**SVC**	0.782 $\pm$ 0.091	0.768 $\pm$ 0.070	0.844 $\pm$ 0.142	0.798 $\pm$ 0.089	0.839 $\pm$ 0.092	**0.812** $\pm$ 0.071	**0.799** $\pm$ 0.087	**0.866** $\pm$ 0.082	**0.827** $\pm$ 0.068	**0.872** $\pm$ 0.076
	**RFC**	**0.791** $\pm$ 0.073	**0.771** $\pm$ 0.081	0.854 $\pm$ 0.084	**0.809** $\pm$ 0.073	**0.857** $\pm$ 0.067	0.788 $\pm$ 0.076	0.758 $\pm$ 0.082	**0.875** $\pm$ 0.099	0.809 $\pm$ 0.074	0.848 $\pm$ 0.084
	**GradBoostC**	0.791 $\pm$ 0.066	0.771 $\pm$ 0.072	**0.854** $\pm$ 0.090	0.808 $\pm$ 0.070	**0.866** $\pm$ 0.076	**0.803** $\pm$ 0.042	**0.787** $\pm$ 0.070	0.853 $\pm$ 0.079	**0.816** $\pm$ 0.057	0.853 $\pm$ 0.067

Table ST1 Comparison of preprocessing strategies. For each model, the best score per metric (Strategy 1 vs. Strategy 2) is marked in bold; in case of ties, the strategy with the lower standard deviation is preferred

Data	Method	Accuracy	Precision	Recall	F1	AUROC
**f-tve**	LogReg (benchmark)	**0.846** ± 0.048	0.902 ± 0.055	**0.886** ± 0.050	0.893 ± 0.042	**0.878** ± 0.052
	LogReg (η=0.1)	0.842 ± 0.040	0.913 ± 0.051	0.867 ± 0.028	**0.889** ± 0.034	0.862 ± 0.059
	LogReg (η=0.2)	0.828 ± 0.044	**0.918** ± 0.049	0.845 ± 0.038	0.879 ± 0.031	0.873 ± 0.063
	SVC (benchmark)	0.864 ± 0.040	**0.897** ± 0.052	0.920 ± 0.053	0.907 ± 0.037	0.882 ± 0.046
	SVC (η=0.1)	0.854 ± 0.043	0.885 ± 0.052	0.920 ± 0.051	0.901 ± 0.038	**0.896** ± 0.044
	SVC (η=0.2)	**0.870** ± 0.030	0.891 ± 0.041	**0.937** ± 0.044	**0.913** ± 0.029	0.895 ± 0.044
	RFC (benchmark)	0.834 ± 0.064	0.872 ± 0.061	0.903 ± 0.056	0.887 ± 0.054	0.856 ± 0.074
	RFC (η=0.1)	**0.848** ± 0.051	0.885 ± 0.054	**0.909** ± 0.048	**0.896** ± 0.044	**0.877** ± 0.061
	RFC (η=0.2)	0.848 ± 0.060	**0.889** ± 0.060	0.904 ± 0.053	0.895 ± 0.049	0.870 ± 0.069
	GradBoostC (benchmark)	0.850 ± 0.051	0.880 ± 0.059	0.920 ± 0.040	0.898 ± 0.043	**0.870** ± 0.078
	GradBoostC (η=0.1)	**0.856** ± 0.036	0.885 ± 0.046	**0.923** ± 0.035	**0.903** ± 0.033	0.861 ± 0.075
	GradBoostC (η=0.2)	0.856 ± 0.037	**0.891** ± 0.045	0.915 ± 0.036	0.902 ± 0.033	0.858 ± 0.065
**f-mth**	LogReg (benchmark)	**0.784** ± 0.051	0.787 ± 0.063	**0.823** ± 0.082	**0.801** ± 0.039	**0.834** ± 0.060
	LogReg (η=0.1)	0.774 ± 0.029	0.781 ± 0.055	0.808 ± 0.077	0.789 ± 0.021	0.825 ± 0.054
	LogReg (η=0.2)	0.768 ± 0.054	**0.790** ± 0.057	0.778 ± 0.091	0.779 ± 0.044	0.826 ± 0.069
	SVC (benchmark)	0.766 ± 0.065	0.758 ± 0.073	0.842 ± 0.112	0.791 ± 0.047	**0.855** ± 0.071
	SVC (η=0.1)	0.782 ± 0.058	0.776 ± 0.075	0.844 ± 0.086	0.803 ± 0.043	0.834 ± 0.082
	SVC (η=0.2)	**0.795** ± 0.062	**0.784** ± 0.078	**0.864** ± 0.078	**0.817** ± 0.043	0.854 ± 0.076
	RFC (benchmark)	**0.789** ± 0.039	0.751 ± 0.050	**0.911** ± 0.078	**0.820** ± 0.029	0.859 ± 0.046
	RFC (η=0.1)	0.787 ± 0.043	**0.787** ± 0.072	0.861 ± 0.091	0.816 ± 0.041	**0.889** ± 0.053
	RFC (η=0.2)	0.782 ± 0.049	0.761 ± 0.060	0.866 ± 0.095	0.806 ± 0.044	0.877 ± 0.051
	GradBoostC (benchmark)	0.758 ± 0.043	0.733 ± 0.057	0.857 ± 0.079	0.787 ± 0.040	0.849 ± 0.047
	GradBoostC (η=0.1)	0.766 ± 0.058	0.740 ± 0.068	0.869 ± 0.053	0.796 ± 0.043	0.866 ± 0.066
	GradBoostC (η=0.2)	**0.800** ± 0.081	**0.782** ± 0.091	**0.880** ± 0.047	**0.825** ± 0.060	**0.866** ± 0.062
**bpu-lac**	LogReg (benchmark)	**0.800** ± 0.062	**0.798** ± 0.072	**0.833** ± 0.074	**0.813** ± 0.058	0.844 ± 0.057
	LogReg (η=0.1)	0.782 ± 0.071	0.774 ± 0.076	0.819 ± 0.109	0.793 ± 0.081	0.843 ± 0.077
	LogReg (η=0.2)	0.776 ± 0.058	0.798 ± 0.082	0.785 ± 0.094	0.785 ± 0.057	**0.850** ± 0.060
	SVC (benchmark)	**0.782** ± 0.091	0.768 ± 0.070	**0.844** ± 0.142	0.798 ± 0.089	0.839 ± 0.092
	SVC (η=0.1)	0.779 ± 0.076	0.773 ± 0.085	0.813 ± 0.113	**0.789** ± 0.083	0.847 ± 0.064
	SVC (η=0.2)	0.776 ± 0.071	**0.789** ± 0.090	0.788 ± 0.094	0.785 ± 0.074	**0.853** ± 0.074
	RFC (benchmark)	0.791 ± 0.073	0.771 ± 0.081	0.854 ± 0.084	0.809 ± 0.073	0.857 ± 0.067
	RFC (η=0.1)	0.774 ± 0.090	0.743 ± 0.093	**0.872** ± 0.086	0.800 ± 0.081	0.829 ± 0.084
	RFC (η=0.2)	**0.800** ± 0.064	**0.785** ± 0.077	0.845 ± 0.095	**0.811** ± 0.074	**0.885** ± 0.051
	GradBoostC (benchmark)	0.791 ± 0.066	0.771 ± 0.072	**0.854** ± 0.090	0.808 ± 0.070	**0.866** ± 0.076
	GradBoostC (η=0.1)	**0.803** ± 0.067	**0.799** ± 0.074	0.821 ± 0.095	**0.809** ± 0.080	0.852 ± 0.072
	GradBoostC (η=0.2)	0.785 ± 0.054	0.786 ± 0.081	0.811 ± 0.077	0.795 ± 0.061	0.862 ± 0.049

Table ST2 Classifier performances on non-augmented vs. GN-augmented data ($\eta = \{0.1, 0.2\}$). For each model, the best scores across the block (banchmark $\eta = \{0.0\}$ vs. $\eta = \{0.1\}$ vs. $\eta = \{0.2\}$) are marked in bold; in case of ties, the strategy with the lower standard deviation is preferred

Data	Method	Accuracy	Precision	Recall	F1	AUROC
**f-tve**	W/o augmentation	0.808 ± 0.056	0.868 ± 0.042	0.873 ± 0.069	0.868 ± 0.044	0.788 ± 0.094
	Augmented	**0.848** ± 0.045	**0.886** ± 0.048	**0.909** ± 0.045	**0.897** ± 0.037	**0.845** ± 0.083
**f-mth**	W/o augmentation	0.760 ± 0.053	0.756 ± 0.054	0.818 ± 0.080	0.782 ± 0.041	0.840 ± 0.058
	Augmented	**0.792** ± 0.070	**0.778** ± 0.070	**0.866** ± 0.064	**0.817** ± 0.048	**0.867** ± 0.049
**bpu-lac**	W/o augmentation	0.779 ± 0.076	**0.789** ± 0.094	0.796 ± 0.088	0.789 ± 0.079	**0.841** ± 0.070
	Augmented	**0.779** ± 0.066	0.786 ± 0.097	**0.798** ± 0.074	**0.789** ± 0.072	0.840 ± 0.063

Table ST3 SMILES augmentation influence on ChemBERTa performance. The best scores across the block (augmented vs. non-augmented data) are marked in bold; in case of ties, lower standard deviation is preferred

Appendix

Theoretical framework for the classical classifiers

Various algorithms may use different objectives (e.g., Gini or entropy for tree-based models). Below, we outline the optimization criteria and loss formulations used by each classifier in our study.

The elastic net logistic regression (LogReg) solves the expected risk minimization (ERM) by minimizing a penalized log-loss:

$$\mathcal{L}\big(y_i, f_\theta(\mathbf{x}_i)\big) = -\frac{1}{n}\sum_{i=1}^{n} \ln(1 + e^{-y_i \theta^T \mathbf{x}_i}) + \lambda\Big[\alpha\|\theta\|_1 + (1-\alpha)\|\theta\|_2^2\Big], \tag{6}$$

where $\|\theta\|_1 = \sum_{j=1}^{p} |\theta_j|$ and $\|\theta\|_2^2 = \sum_{j=1}^{p} \theta_j^2$ are the Lasso (L_1) and Ridge (L_2) penalties, $\lambda > 0$ controls regularization strength, $\alpha \in [0, 1]$ interpolates between pure Lasso ($\alpha = 1$) and Ridge ($\alpha = 0$). Elastic net encourages both sparsity and stability, which can be beneficial when working with high-dimensional, potentially collinear chemical descriptors.

The support vector classifier (SVC) searches for a hyperplane in the feature space that maximizes the margin between classes while allowing soft penalties for misclassification. Its soft-margin objective is:

$$\mathcal{L}\big(y_i, f_\theta(\mathbf{x}_i)\big) = \|\theta\|_2^2 + C\sum_{i=1}^{n} \max\big(0, 1 - y_i\theta^T\mathbf{x}_i\big), \tag{7}$$

where $C > 0$ controls the trade-off between margin maximization and classification error minimization, the hinge loss term $\max\big(0, 1 - y_i\theta^T\mathbf{x}_i\big)$ penalizes data points $\mathbf{x}_i$ that fall inside the margin or on the wrong side of it.

The above formulation is the primal form of the soft-margin SVC and extends naturally to nonlinear decision boundaries via kernel functions [93].

The random forest classifier (RFC) is an ensemble method that builds multiple decision trees, each trained on a bootstrapped subset of the training data. For classification, the final prediction for point i is obtained by aggregating the outputs of the individual trees $t = 1, \ldots, T$, typically via majority voting:

$$f(\mathbf{x}_i) = \text{majority}\big\{h_t(\mathbf{x}_i)\big\}_{t=1}^{T}, \tag{8}$$

where h_t is the prediction of the t-th tree.

Each tree is grown through recursive binary splits based on descriptor thresholds to minimize a local impurity. We use Gini impurity criterion[7]. For a node g with data $S_g \subset \mathcal{D}$, a binary split on a feature j at threshold τ yields two (left and right) child nodes:

$$S_g^L = \big\{(x_i, y_i) \in S_g \mid x_{i,j} \le \tau\big\}, \quad S_g^R = \big\{(x_i, y_i) \in S_g \mid x_{i,j} > \tau\big\}. \tag{9}$$

[7] Default choice in the `sklearn.RandomForestClassifier()` class.

The optimal split minimizes the impurity-based risk:

$$\mathcal{L}_{\text{split}}(S_g) = \frac{|S_g^L|}{|S_g|}\text{impurity}(S_g^L) + \frac{|S_g^R|}{|S_g|}\text{impurity}(S_g^R), \tag{10}$$

where $|\cdot|$ denotes the cardinality of a set. For binary classification, the Gini impurity $\mathcal{G}$ for split S_g is:

$$\mathcal{G}(S_g) = 1 - \left(p_g^2 + (1-p_g)^2\right), \quad p_g = \frac{1}{|S_g|}\sum_{(x_i,y_i)\in S_g} \mathbf{1}_{\{y_i=1\}}. \tag{11}$$

While RFC does not directly minimize a global loss like LogReg or SVC, it implicitly minimizes the empirical risk across the forest via greedy local impurity reduction. An ensemble-level approximation of the impurity-based loss is:

$$\mathcal{L}(S) = \frac{1}{T}\sum_{t=1}^{T}\sum_{g\in h_t}\left(\frac{|S_g|}{n}\mathcal{G}(S_g)\right). \tag{12}$$

The gradient boosting classifier (GradBoostC) builds an additive ensemble of decision trees in a stage-wise manner, with each new tree trained to correct the residual errors made by the current ensemble, essentially approximating the solution to the ERM problem defined in (1) using a functional gradient descent strategy.

The prediction function is represented as an ensemble of M weighted trees:

$$f_\theta(\mathbf{x}) = \sum_{m=1}^{M} \gamma^{(m)} h^{(m)}(\mathbf{x}), \tag{13}$$

where each weak learner $h^{(m)}$ is a tree that partitions the input space into $J^{(m)}$ disjoint regions $\{R_{J^{(m)}}^{(m)}\}_{j=1}^{J^{(m)}}$ with a constant prediction $b_j^{(m)}$ in the region $R_j^{(m)}$:

$$h^{(m)}(\mathbf{x}) = \sum_{j=1}^{J^{(m)}} b_j^{(m)} \mathbf{1}_{\{R_j^{(m)}(\mathbf{x})\}}. \tag{14}$$

At each iteration $m = 1, \ldots, M$, GradBoostC computes the pseudo residuals as the negative gradient of the loss with respect to the current predictions:

$$r_i^{(m)} = -\frac{\partial \mathcal{L}\big(y_i, f_\theta(\mathbf{x}_i)\big)}{\partial f_\theta(\mathbf{x}_i)}\Bigg|_{f_\theta(\mathbf{x}) = f_\theta^{(m-1)}(\mathbf{x})} =^{8} \frac{2y_i}{1+e^{2y_i f_\theta^{(m-1)}(\mathbf{x}_i)}}.$$

[8] Given a smoothed exponential loss function $\mathcal{L}(y_i, f_\theta(\mathbf{x}_i) = \ln(1 + e^{-2y_i f_\theta(\mathbf{x}_i)}))$ used in the AdaBoost optimization of the `sklearn.GradientBoostingClassifier()` class.

The learner is then trained on the pseudo residuals $\{(\mathbf{x}_i, r_i^{(m)})\}_{i=1}^{n}$, and the ensemble is updated as:

$$f_\theta^{(m)}(\mathbf{x}) = f_\theta^{(m-1)}(\mathbf{x}) + \gamma^{(m)} h^{(m)}(\mathbf{x}). \tag{15}$$

The learning rate γ_m is determined via line search:

$$\gamma^{(m)} = \operatorname*{argmin}_{\gamma} \sum_{i=1}^{n} \mathcal{L}\big(y_i, f_\theta^{(m-1)}(\mathbf{x}_i) + \gamma h^{(m)}(\mathbf{x}_i)\big).$$

This stage-wise strategy progressively refines the model's predictions, converging to a function that minimizes classification loss.

References

[1] Claus, H., Filip, Z.: Degradation and transformation of aquatic humic substances by laccase-producing fungi cladosporium cladosporioides and polyporus versicolor. Acta hydrochimica et hydrobiologica **26**(3), 180–185 (1998)

[2] Ihssen, J., Reiss, R., Luchsinger, R., Thöny-Meyer, L., Richter, M.: Biochemical properties and yields of diverse bacterial laccase-like multicopper oxidases expressed in escherichia coli. Scientific reports **5**(1), 10465 (2015)

[3] Su, J., Fu, J., Wang, Q., Silva, C., Cavaco-Paulo, A.: Laccase: a green catalyst for the biosynthesis of poly-phenols. Critical Reviews in Biotechnology **38**(2), 294–307 (2018)

[4] Brugnari, T., Braga, D.M., Dos Santos, C.S.A., Torres, B.H.C., Modkovski, T.A., Haminiuk, C.W.I., Maciel, G.M.: Laccases as green and versatile biocatalysts: from lab to enzyme market—an overview. Bioresources and Bioprocessing **8**, 1–29 (2021)

[5] Mahuri, M., Paul, M., Thatoi, H.: A review of microbial laccase production and activity toward different biotechnological applications. Systems Microbiology and Biomanufacturing **3**(4), 533–551 (2023)

[6] Meyer, H.-P., Eichhorn, E., Hanlon, S., Lütz, S., Schürmann, M., Wohlgemuth, R., Coppolecchia, R.: The use of enzymes in organic synthesis and the life sciences: perspectives from the swiss industrial biocatalysis consortium (sibc). Catalysis Science & Technology **3**(1), 29–40 (2013)

[7] Pezzella, C., Guarino, L., Piscitelli, A.: How to enjoy laccases. Cellular and Molecular Life Sciences **72**, 923–940 (2015)

[8] Claus, H., Faber, G., König, H.: Redox-mediated decolorization of synthetic dyes by fungal laccases. Applied microbiology and biotechnology **59**, 672–678 (2002)

[9] Camarero, S., Ibarra, D., Martinez, M.J., Martinez, A.T.: Lignin-derived compounds as efficient laccase mediators for decolorization of different types of recalcitrant dyes. Applied and environmental microbiology **71**(4), 1775–1784 (2005)

[10] Minussi, R.C., Pastore, G.M., Durán, N.: Potential applications of laccase in the food industry. Trends in Food Science & Technology **13**(6-7), 205–216 (2002)

[11] Battista, E., Lettera, V., Villani, M., Calestani, D., Gentile, F., Netti, P.A., Iannotta, S., Zappettini, A., Coppedè, N.: Enzymatic sensing with laccase-functionalized textile organic biosensors. Organic electronics **40**, 51–57 (2017)

[12] Duran, N., Esposito, E.: Potential applications of oxidative enzymes and phenoloxidase-like compounds in wastewater and soil treatment: a review. Applied

catalysis B: environmental **28**(2), 83–99 (2000)

[13] Arregui, L., Ayala, M., Gómez-Gil, X., Gutiérrez-Soto, G., Hernández-Luna, C.E., Herrera De Los Santos, M., Levin, L., Rojo-Domínguez, A., Romero-Martínez, D., Saparrat, M.C., *et al.*: Laccases: structure, function, and potential application in water bioremediation. Microbial cell factories **18**, 1–33 (2019)

[14] Strong, P., Claus, H.: Laccase: a review of its past and its future in bioremediation. Critical Reviews in Environmental Science and Technology **41**(4), 373–434 (2011)

[15] Datta, S., Veena, R., Samuel, M.S., Selvarajan, E.: Immobilization of laccases and applications for the detection and remediation of pollutants: a review. Environmental Chemistry Letters **19**(1), 521–538 (2021)

[16] Widsten, P., Kandelbauer, A.: Laccase applications in the forest products industry: a review. Enzyme and microbial technology **42**(4), 293–307 (2008)

[17] Schubert, M., Engel, J., Thöny-Meyer, L., Schwarze, F., Ihssen, J.: Protection of wood from microorganisms by laccase-catalyzed iodination. Applied and environmental microbiology **78**(20), 7267–7275 (2012)

[18] Ihssen, J., Schubert, M., Thöny-Meyer, L., Richter, M.: Laccase catalyzed synthesis of iodinated phenolic compounds with antifungal activity. PLoS One **9**(3), 89924 (2014)

[19] Jankowska, D., Heck, T., Schubert, M., Yerlikaya, A., Weymuth, C., Rentsch, D., Schober, I., Richter, M.: Enzymatic synthesis of lignin-based concrete dispersing agents. ChemBioChem **19**(13), 1365–1369 (2018)

[20] Ray, S., Kumar, R.: A snapshot review on enzymatic modification of wood. MRS Advances **7**(27), 562–569 (2022)

[21] Reiss, R., Ihssen, J., Richter, M., Eichhorn, E., Schilling, B., Thöny-Meyer, L.: Laccase versus laccase-like multi-copper oxidase: a comparative study of similar enzymes with diverse substrate spectra. PloS one **8**(6), 65633 (2013)

[22] Jones, S.M., Solomon, E.I.: Electron transfer and reaction mechanism of laccases. Cellular and molecular life sciences **72**, 869–883 (2015)

[23] Claus, H.: Laccases: structure, reactions, distribution. Micron **35**(1-2), 93–96 (2004)

[24] Irwin, J.J., Shoichet, B.K.: Zinc- a free database of commercially available compounds for virtual screening. Journal of chemical information and modeling **45**(1), 177–182 (2005)

[25] Wishart, D.S., Knox, C., Guo, A.C., Shrivastava, S., Hassanali, M., Stothard, P., Chang, Z., Woolsey, J.: Drugbank: a comprehensive resource for in silico drug

discovery and exploration. Nucleic acids research **34**(suppl_1), 668–672 (2006)

[26] Gaulton, A., Bellis, L.J., Bento, A.P., Chambers, J., Davies, M., Hersey, A., Light, Y., McGlinchey, S., Michalovich, D., Al-Lazikani, B., *et al.*: Chembl: a large-scale bioactivity database for drug discovery. Nucleic acids research **40**(D1), 1100–1107 (2012)

[27] Kim, S., Thiessen, P.A., Bolton, E.E., Chen, J., Fu, G., Gindulyte, A., Han, L., He, J., He, S., Shoemaker, B.A., *et al.*: Pubchem substance and compound databases. Nucleic acids research **44**(D1), 1202–1213 (2016)

[28] Sakiyama, Y.: The use of machine learning and nonlinear statistical tools for adme prediction. Expert opinion on drug metabolism & toxicology **5**(2), 149–169 (2009)

[29] Feinberg, E.N., Sur, D., Wu, Z., Husic, B.E., Mai, H., Li, Y., Sun, S., Yang, J., Ramsundar, B., Pande, V.S.: Potentialnet for molecular property prediction. ACS central science **4**(11), 1520–1530 (2018)

[30] Tao, L., Zhang, P., Qin, C., Chen, S., Zhang, C., Chen, Z., Zhu, F., Yang, S., Wei, Y., Chen, Y.: Recent progresses in the exploration of machine learning methods as in-silico adme prediction tools. Advanced Drug Delivery Reviews **86**, 83–100 (2015)

[31] Shilpa, S., Kashyap, G., Sunoj, R.B.: Recent applications of machine learning in molecular property and chemical reaction outcome predictions. The Journal of Physical Chemistry A **127**(40), 8253–8271 (2023)

[32] Wieder, O., Kohlbacher, S., Kuenemann, M., Garon, A., Ducrot, P., Seidel, T., Langer, T.: A compact review of molecular property prediction with graph neural networks. Drug Discovery Today: Technologies **37**, 1–12 (2020)

[33] Walters, W.P., Barzilay, R.: Applications of deep learning in molecule generation and molecular property prediction. Accounts of chemical research **54**(2), 263–270 (2020)

[34] Heid, E., Greenman, K.P., Chung, Y., Li, S.-C., Graff, D.E., Vermeire, F.H., Wu, H., Green, W.H., McGill, C.J.: Chemprop: a machine learning package for chemical property prediction. Journal of Chemical Information and Modeling **64**(1), 9–17 (2023)

[35] Méndez-Lucio, O., Nicolaou, C.A., Earnshaw, B.: Mole: a foundation model for molecular graphs using disentangled attention. Nature Communications **15**(1), 9431 (2024)

[36] Schneider, G., Wrede, P.: Artificial neural networks for computer-based molecular design. Progress in biophysics and molecular biology **70**(3), 175–222 (1998)

[37] Sanchez-Lengeling, B., Aspuru-Guzik, A.: Inverse molecular design using machine learning: Generative models for matter engineering. Science **361**(6400), 360–365 (2018)

[38] Elton, D.C., Boukouvalas, Z., Fuge, M.D., Chung, P.W.: Deep learning for molecular design—a review of the state of the art. Molecular Systems Design & Engineering **4**(4), 828–849 (2019)

[39] Brown, N., Fiscato, M., Segler, M.H., Vaucher, A.C.: Guacamol: benchmarking models for de novo molecular design. Journal of chemical information and modeling **59**(3), 1096–1108 (2019)

[40] Sousa, T., Correia, J., Pereira, V., Rocha, M.: Generative deep learning for targeted compound design. Journal of chemical information and modeling **61**(11), 5343–5361 (2021)

[41] Ivanenkov, Y.A., Polykovskiy, D., Bezrukov, D., Zagribelnyy, B., Aladinskiy, V., Kamya, P., Aliper, A., Ren, F., Zhavoronkov, A.: Chemistry42: an ai-driven platform for molecular design and optimization. Journal of chemical information and modeling **63**(3), 695–701 (2023)

[42] Meuwly, M.: Machine learning for chemical reactions. Chemical Reviews **121**(16), 10218–10239 (2021)

[43] Ranković, B., Schwaller, P.: Bochemian: Large language model embeddings for bayesian optimization of chemical reactions. In: NeurIPS 2023 Workshop on Adaptive Experimental Design and Active Learning in the Real World (2023)

[44] Zhang, D., Liu, W., Tan, Q., Chen, J., Yan, H., Yan, Y., Li, J., Huang, W., Yue, X., Ouyang, W., et al.: Chemllm: A chemical large language model. arXiv preprint arXiv:2402.06852 (2024)

[45] Ramos, M.C., Collison, C.J., White, A.D.: A review of large language models and autonomous agents in chemistry. Chemical Science (2025)

[46] Probst, D., Manica, M., Nana Teukam, Y.G., Castrogiovanni, A., Paratore, F., Laino, T.: Biocatalysed synthesis planning using data-driven learning. Nature communications **13**(1), 964 (2022)

[47] Yu, T., Boob, A.G., Volk, M.J., Liu, X., Cui, H., Zhao, H.: Machine learning-enabled retrobiosynthesis of molecules. Nature Catalysis **6**(2), 137–151 (2023)

[48] Casadevall, G., Duran, C., Osuna, S.: Alphafold2 and deep learning for elucidating enzyme conformational flexibility and its application for design. JACS Au **3**(6), 1554–1562 (2023)

[49] Jiang, Y., Ran, X., Yang, Z.J.: Data-driven enzyme engineering to identify

function-enhancing enzymes. Protein Engineering, Design and Selection **36**, 009 (2023)

[50] Rogers, D., Hahn, M.: Extended-connectivity fingerprints. Journal of chemical information and modeling **50**(5), 742–754 (2010)

[51] Bajusz, D., Rácz, A., Héberger, K.: Why is tanimoto index an appropriate choice for fingerprint-based similarity calculations? Journal of cheminformatics **7**(1), 20 (2015)

[52] McInnes, L., Healy, J., Melville, J.: Umap: Uniform manifold approximation and projection for dimension reduction. arXiv preprint arXiv:1802.03426 (2018)

[53] Bemis, G.W., Murcko, M.A.: The properties of known drugs. 1. molecular frameworks. Journal of medicinal chemistry **39**(15), 2887–2893 (1996)

[54] Wigh, D.S., Goodman, J.M., Lapkin, A.A.: A review of molecular representation in the age of machine learning. Wiley Interdisciplinary Reviews: Computational Molecular Science **12**(5), 1603 (2022)

[55] Yang, K., Swanson, K., Jin, W., Coley, C., Eiden, P., Gao, H., Guzman-Perez, A., Hopper, T., Kelley, B., Mathea, M., *et al.*: Analyzing learned molecular representations for property prediction. Journal of chemical information and modeling **59**(8), 3370–3388 (2019)

[56] Deng, J., Yang, Z., Wang, H., Ojima, I., Samaras, D., Wang, F.: A systematic study of key elements underlying molecular property prediction. Nature Communications **14**(1), 6395 (2023)

[57] Dalby, A., Nourse, J.G., Hounshell, W.D., Gushurst, A.K., Grier, D.L., Leland, B.A., Laufer, J.: Description of several chemical structure file formats used by computer programs developed at molecular design limited. Journal of chemical information and computer sciences **32**(3), 244–255 (1992)

[58] David, L., Thakkar, A., Mercado, R., Engkvist, O.: Molecular representations in ai-driven drug discovery: a review and practical guide. Journal of cheminformatics **12**(1), 56 (2020)

[59] Liu, S., Wang, H., Liu, W., Lasenby, J., Guo, H., Tang, J.: Pre-training molecular graph representation with 3d geometry. arXiv preprint arXiv:2110.07728 (2021)

[60] Zhu, J., Xia, Y., Wu, L., Xie, S., Qin, T., Zhou, W., Li, H., Liu, T.-Y.: Unified 2d and 3d pre-training of molecular representations. In: Proceedings of the 28th ACM SIGKDD Conference on Knowledge Discovery and Data Mining, pp. 2626–2636 (2022)

[61] Weininger, D.: Smiles, a chemical language and information system. 1. introduction to methodology and encoding rules. Journal of chemical information and computer sciences **28**(1), 31–36 (1988)

[62] Daylight Chemical Information Systems, I.: Smarts-a language for describing molecular patterns (2019)

[63] Krenn, M., Ai, Q., Barthel, S., Carson, N., Frei, A., Frey, N.C., Friederich, P., Gaudin, T., Gayle, A.A., Jablonka, K.M., et al.: Selfies and the future of molecular string representations. Patterns **3**(10) (2022)

[64] Mauri, A., Consonni, V., Pavan, M., Todeschini, R., *et al.*: Dragon software: An easy approach to molecular descriptor calculations. Match **56**(2), 237–248 (2006)

[65] Shalev-Shwartz, S., Ben-David, S.: Understanding Machine Learning: From Theory to Algorithms. Cambridge university press, ??? (2014)

[66] Chithrananda, S., Grand, G., Ramsundar, B.: Chemberta: large-scale self-supervised pretraining for molecular property prediction. arXiv preprint arXiv:2010.09885 (2020)

[67] Ahmad, W., Simon, E., Chithrananda, S., Grand, G., Ramsundar, B.: Chemberta-2: Towards chemical foundation models. arXiv preprint arXiv:2209.01712 (2022)

[68] Liu, Y., Ott, M., Goyal, N., Du, J., Joshi, M., Chen, D., Levy, O., Lewis, M., Zettlemoyer, L., Stoyanov, V.: Roberta: A robustly optimized bert pretraining approach. arXiv preprint arXiv:1907.11692 (2019)

[69] PyTorch Contributors: torch.nn.BCELoss — PyTorch documentation. Accessed: 2025-07-07 (2024). https://pytorch.org/docs/stable/generated/torch.nn.BCELoss.html

[70] Pedregosa, F., Varoquaux, G., Gramfort, A., Michel, V., Thirion, B., Grisel, O., Blondel, M., Prettenhofer, P., Weiss, R., Dubourg, V., Vanderplas, J., Passos, A., Cournapeau, D., Brucher, M., Perrot, M., Duchesnay, É.: Scikit-learn: Machine learning in Python. Journal of Machine Learning Research **12**, 2825–2830 (2011)

[71] Wolf, T., Debut, L., Sanh, V., Chaumond, J., Delangue, C., Moi, A., Cistac, P., Rault, T., Louf, R., Funtowicz, M., *et al.*: Transformers: State-of-the-art natural language processing. In: Proceedings of the 2020 Conference on Empirical Methods in Natural Language Processing: System Demonstrations, pp. 38–45 (2020)

[72] Paszke, A., Gross, S., Massa, F., Lerer, A., Bradbury, J., Chanan, G., Killeen, T., Lin, Z., Gimelshein, N., Antiga, L., *et al.*: Pytorch: An imperative style, high-performance deep learning library. In: Advances in Neural Information Processing Systems, vol. 32 (2019). Curran Associates, Inc.

[73] Akiba, T., Sano, S., Yanase, T., Ohta, T., Koyama, M.: Optuna: A next-generation hyperparameter optimization framework. In: Proceedings of the 25th ACM SIGKDD International Conference on Knowledge Discovery & Data Mining, pp. 2623–2631 (2019)

[74] Chawla, N.V., Bowyer, K.W., Hall, L.O., Kegelmeyer, W.P.: Smote: synthetic minority over-sampling technique. Journal of artificial intelligence research **16**, 321–357 (2002)

[75] Han, H., Wang, W.-Y., Mao, B.-H.: Borderline-smote: a new over-sampling method in imbalanced data sets learning. In: International Conference on Intelligent Computing, pp. 878–887 (2005). Springer

[76] LemaÃŽtre, G., Nogueira, F., Aridas, C.K.: Imbalanced-learn: A python toolbox to tackle the curse of imbalanced datasets in machine learning. Journal of machine learning research **18**(17), 1–5 (2017)

[77] Friedman, J.H.: Greedy function approximation: a gradient boosting machine. Annals of statistics, 1189–1232 (2001)

[78] Domingos, P.: A few useful things to know about machine learning. Communications of the ACM **55**(10), 78–87 (2012)

[79] Shwartz-Ziv, R., Armon, A.: Tabular data: Deep learning is not all you need. Information Fusion **81**, 84–90 (2022)

[80] Borisov, V., Leemann, T., Seßler, K., Haug, J., Pawelczyk, M., Kasneci, G.: Deep neural networks and tabular data: A survey. IEEE transactions on neural networks and learning systems (2022)

[81] Srivastava, N., Hinton, G., Krizhevsky, A., Sutskever, I., Salakhutdinov, R.: Dropout: a simple way to prevent neural networks from overfitting. The journal of machine learning research **15**(1), 1929–1958 (2014)

[82] Bertrand, T., Jolivalt, C., Briozzo, P., Caminade, E., Joly, N., Madzak, C., Mougin, C.: Crystal structure of a four-copper laccase complexed with an arylamine: insights into substrate recognition and correlation with kinetics. Biochemistry **41**(23), 7325–7333 (2002)

[83] Pardo, I., Camarero, S.: Laccase engineering by rational and evolutionary design. Cellular and molecular life sciences **72**, 897–910 (2015)

[84] Ankan, A., Textor, J.: pgmpy: A python toolkit for bayesian networks. Journal of Machine Learning Research **25**(265), 1–8 (2024)

[85] Fayyad, U.M., Irani, K.B.: Multi-interval discretization of continuous-valued attributes for classification learning. In: Ijcai, vol. 93, pp. 1022–1029 (1993)

[86] Vig, J.: Bertviz: A tool for visualizing multihead self-attention in the bert model. In: ICLR Workshop: Debugging Machine Learning Models, vol. 3 (2019)

[87] Cortes-Ciriano, I., Bender, A.: Improved chemical structure–activity modeling through data augmentation. Journal of chemical information and modeling **55**(12), 2682–2692 (2015)

[88] Shorten, C., Khoshgoftaar, T.M.: A survey on image data augmentation for deep learning. Journal of big data **6**(1), 1–48 (2019)

[89] Xu, L., Skoularidou, M., Cuesta-Infante, A., Veeramachaneni, K.: Modeling tabular data using conditional gan. Advances in neural information processing systems **32** (2019)

[90] Shorten, C., Khoshgoftaar, T.M., Furht, B.: Text data augmentation for deep learning. Journal of big Data **8**(1), 101 (2021)

[91] Moles, L., Andres, A., Echegaray, G., Boto, F.: Exploring data augmentation and active learning benefits in imbalanced datasets. Mathematics **12**(12), 1898 (2024)

[92] Wang, Z., Wang, P., Liu, K., Wang, P., Fu, Y., Lu, C.-T., Aggarwal, C.C., Pei, J., Zhou, Y.: A comprehensive survey on data augmentation. arXiv preprint arXiv:2405.09591 (2024)

[93] Schölkopf, B., Smola, A.J.: Learning with Kernels: Support Vector Machines, Regularization, Optimization, and Beyond. MIT press, ??? (2002)
